# Chlorination: An Effective Strategy for High‐Performance Organic Solar Cells

**DOI:** 10.1002/advs.202000509

**Published:** 2020-06-09

**Authors:** Qiaoqiao Zhao, Jianfei Qu, Feng He

**Affiliations:** ^1^ Shenzhen Grubbs Institute and Department of Chemistry Southern University of Science and Technology Shenzhen 518055 China; ^2^ Guangdong Provincial Key Laboratory of Catalysis Southern University of Science and Technology Shenzhen 518055 China

**Keywords:** 3d orbital, chlorination, electronegativity, energy levels, solar cells

## Abstract

This work summarizes recent developments in polymer solar cells (PSCs) prepared by a chlorination strategy. The intrinsic property of chlorine atoms, the progress of chlorinated polymers and small molecules, and the synergistic effect of chlorination with other methods to elevate solar conversions are discussed. Halogenation of donor–acceptor (D–A) materials is an effective method to improve the performance of PSCs, which mainly affects the push–pull of electrons between donor and acceptor units due to their strong electron‐withdrawing capabilities. Although chlorine is less electronegative than fluorine, it can form very strong noncovalent interactions, such as Cl···S and Cl···*π* interactions, because its empty 3d orbits can help to accept the electron pairs or *π* electrons. This synergistic effect of electronegativity together with the empty 3d orbits of chlorine atoms leads to increased intramolecular and intermolecular interactions and a much stronger capability to down‐shift the molecular energy levels. This work is intended to support a better understanding of the chlorination strategy to modify the material properties, and thus improve the performance of solar devices. Eventually, it will provide the research community with a clearer pathway to choose proper substitution methods according to different situations for high and stable solar energy conversion.

## Graphical abstract

Halogenation of D–A (donor–acceptor)‐type materials is an effective method to improve the performance of polymer solar cells (PSCs). In this work, recent developments of PSCs by the chlorination strategy are summarized, including the intrinsic property of chlorine atoms, the progress of chlorinated polymers and small molecules, and the synergetic effect of chlorination with other methods to elevate solar conversions.

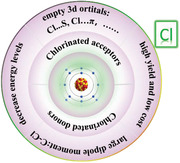

## 1 Introduction

Recently, organic solar cells (OSC) have been subject to rapid development. Many excellent OSCs with high power conversion efficiency (PCE) have been fabricated,^[^ ^1^, ^2^, ^3^, ^4^, ^5^, ^6^ ^]^ and the highest PCE in OSCs is already over 18%.^[^ ^1^ ^]^ This brings OSCs closer to becoming the next generation of applicable solar cells with the advantages of lower cost and better lightweight characteristics and flexibility than inorganic solar cells. Enhancements of PCE lie mainly in the molecular structure design.

Improved donor–acceptor‐type copolymers have in recent years become the main research goal of new conjugated polymer donor materials for polymer solar cells (PSC). Efficient polymer donor materials require broad visible‐near‐infrared absorption and low highest occupied molecular orbital (HOMO) energy levels, both of which can be achieved by appropriate D–A copolymerization of donor and acceptor structural units. This is the main reason why D–A copolymers are the main research object of new conjugated polymer donor materials. The bandgap of polymer materials is affected by their main‐chain structure, side‐chain structure, and chain interaction. The most important methods to reduce the bandgap of the polymer include introduction of alternating D–A units and quinone structure. For the D–A polymer, intramolecular charge transfer (IACT) occurs due to the push–pull of electrons between the unit D and the unit A, which reduces the bandgap of the polymer and enables it to absorb sunlight at longer wavelengths. In addition, D–A copolymers often have two absorption peaks, the absorption of the conjugated main chain and that of the IACT in the direction of longer wavelengths. Consequently, D–A copolymers have a wide absorption band in the visible‐near‐infrared region. All these factors are conducive to improvement of the utilization of sunlight. For the electron level, the HOMO level of a D–A copolymer depends mainly on the HOMO level of the donor unit, while the lowest unoccupied molecular orbital (LUMO) level depends mainly on the LUMO level of the donor cell. Selection of appropriate donor and acceptor units can therefore easily regulate the HOMO and LUMO energy levels of copolymers. This is also an important means by which photovoltaic devices can improve open‐circuit voltage (*V*_OC_). In the molecular design of D–A copolymer photovoltaic materials, flexible substituents are also needed to improve the solubility of polymers. At the same time, a *π*‐bridge, such as a thiophene unit between the D and A units is needed to reduce the steric hindrance and improve the molecular planarity. In the early years, D–A copolymers were designed and synthesized for electroluminescent polymers with balanced electron and hole polarity^[^ ^7^, ^8^ ^]^ or red‐emitting copolymers with a narrow bandgap.^[^ ^9^ ^]^ In 2003, Andersson et al. first applied the PFDTBT to the PSCs and obtained a PCE of 2.4%.^[^ ^10^ ^]^ After that, several other D–A copolymers were designed and synthesized, promoting the rapid development of PSCs.

Halogenation of a D–A copolymer is an effective method to further improve the performance of PSCs. It mainly affects the push–pull of electrons between unit D and unit A, because typically, a halogen substituent is strong electron‐withdrawing group. The details of the effects depend upon the variety of the halogens, the location of halogenation and the actual structure of the copolymers. Fluorination and chlorination are two main kinds of halogenation and of these, fluorination is a mature approach, while chlorination is a relatively new method and also the key subject of this review.

We will begin by introducing the intrinsic properties of the chlorine atom and compare it with those of the fluorine atom. This is followed by an introduction into the progress of chlorinated polymers and small molecule acceptors. Then, we will summarize the characteristics of chlorination and compare such chlorinated compounds with their fluorinated counterparts, thus providing a straightforward understanding of different halogenation patterns in highly efficient polymer solar cells. Finally, we will compare the synergetic effect of chlorination with other methods to elevate the performance of solar devices.

## 2 Intrinsic Properties of Chlorine and Fluorine

Fluorination is a mature and popular strategy and many reviews have summarized the effect of fluorination on the performance of OSCs. Chlorination on the other hand is an emerging strategy and enhanced device performance with chlorination has been reported infrequently. Because its outstanding performance results from its unique intrinsic properties, we will elaborate on the related basic properties of chlorine and those of fluorine, with the aim of deepening the understanding of chlorination. We will then compare chlorination to fluorination and other substitution.

### 2.1 The Electronegativity of Chlorine and Fluorine

With a Pauling electronegativity of 4.0, the fluorine atom is the strongest electronegative element in the periodic table while the Pauling electronegativity of chlorine at 3.2, is a little lower.^[^ ^11^ ^]^ For D–A polymers, IACT is key to the performance, which occurs due to the push–pull of electrons between unit D and unit A. The introduction of fluorine or/and chlorine with strong electronegativity can affect the electron density distribution along the conjugated backbone of the copolymer base, leading to lower energy levels, usually in both the LUMO and HOMO levels. Both fluorination and chlorination have the potential to decrease both the LUMO and HOMO levels. As expected, bulk‐heterojunction (BHJ) PSCs based on these fluorinated molecules display higher *V*_OC_ and PCE compared to the corresponding nonfluorinated molecules.^[^ ^12^, ^13^ ^]^ Unexpectedly, chlorination usually deepens the energy levels much more efficiently than fluorination, although chlorine is less electronegative, a point which will be discussed in a later section. Due to the strongest electronegativity, many intermolecular or intramolecular noncovalent interactions involve fluorine, such as C—F···H or F···S.^[^ ^14^, ^15^ ^]^ The intermolecular noncovalent interactions are helpful for molecular aggregation, while the intramolecular noncovalent interactions are beneficial for the improvement of molecular planarity.

### 2.2 The van der Waals Radii of Chlorine and Fluorine

Fluorine has a small van der Waals radius of 1.47 Å, while the van der Waals radius of chlorine is 1.76 Å.^[^ ^16^, ^17^ ^]^ Addition of fluorine to a molecule generally fails to lead to serious steric hindrance and with its small size, fluorine tends to retain the planarity of copolymer. The small size of the fluorine atom could lead to severe electrostatic repulsion for *π*‐electrons delocalized in its vicinity.^[^ ^18^ ^]^ Since chlorine has a larger atomic radius than F, the effect of chlorination on intermolecular packing should be considered carefully and the situation for chlorination is more complicated. With its larger atomic radius, the introduction of a chlorine atom will produce larger steric hindrance than fluorine and distort the molecular structure, leading to the damage of IACT, which is seen in some cases. However, the opposite is also true in some cases due to the empty 3d orbital of chlorine, which will be discussed in the following Section.

### 2.3 The Atomic Orbitals of Chlorine and Fluorine

The empty orbit adjacent to 2p of fluorine is 3s. However, the 3s orbit is prohibitively high in energy,^[^ ^18^ ^]^ leading to absence of energetically accessible empty orbits into which the fluorine atom can accept electrons. Chlorine has an empty 3d orbit, which can accept electron pairs and/or *π* electrons, profoundly affecting the property of chlorinated organic semiconductors. Although a Cl atom is less electronegative than an F atom, its empty 3d orbit can help to accept the electron pairs and *π* electrons, which in turn can help to form strong noncovalent interactions, such as Cl···S and Cl···*π*, and can also result in stronger ability to decrease the molecular energy levels. For example, in donors such as PBDB‐T‐2F versus PBDB‐T‐2Cl and acceptors IT‐4F versus IT‐4Cl, chlorinated molecules show lower energy levels than the corresponding fluorinated molecules.^[^ ^19^, ^20^ ^]^

### 2.4 Dipole Moments of the C—Cl and C—F Bonds

The large dipole moment of the C—Cl bond is a feature of chlorinated materials. Hou et al. found that the replacement of F by Cl increased the dipole moments of end groups from 2.26 to 2.77 D.^[^ ^19^ ^]^ A large dipole moment will enhance the IACT effect between D–A units,^[^ ^19^, ^21^ ^]^ and thus expand the absorption spectrum, deepen the molecular energy levels and facilitate intermolecular stacking, which also leads to expansion of the absorption spectra.

## 3 Chlorinated Materials for PSC

At an early stage, chlorination was used to synthesize organic semiconductor materials for organic field effect transistors (OFET) with high and balanced ambipolar transport, because the chlorine atom deepens the LUMO energy level and decreases the charge injection barrier for electrons, making it possible that the electron and the hole can transport simultaneously. Bao et al. found that if chlorination did not break the planarity of the conjugated core, the chlorinated molecules usually had a slightly smaller gap between HOMO and LUMO levels and a lower LUMO than the corresponding fluorinated molecules, probably because Cl has empty 3d orbitals to accommodate *π*‐electrons from the conjugated core, while fluorine atom does not contain accessible empty orbitals for such delocalization.^[^ ^18^ ^]^ Since 2015, chlorination has drawn increasing attention in the application of OSCs.^[^ ^22^, ^23^ ^]^ In only 5 years, the PCE of chlorination‐based OSCs has been improved from only 1% to about 18%.^[^ ^24^, ^25^ ^]^

### 3.1 The Progress of Chlorinated Polymer Donors

Chlorination of polymer donors is an effective strategy with which to increase *V*_OC_, because the introduction of a chlorine atom with strong electronegativity and ability to accommodate delocalized electrons can efficiently deepen the HOMO and LUMO levels and the *V*_OC_ is determined by the gap between HOMO of the donor and LUMO of the acceptor. D–A copolymer photovoltaic materials usually consist of a D unit, an A unit and flexible substituents to improve the solubility of the polymers. A *π*‐bridge between D and A units is sometimes needed to reduce the steric hindrance and improve the molecular planarity. In some cases, a conjugated side chain is added to enhance the coplanarity of the main chain, consequently expanding the absorption band to longer wavelengths.^[^ ^26^ ^]^ As the research into chlorination continues, chlorine atoms have been introduced into the D unit, the A unit, the *π*‐bridge and conjugated side chains. These are summarized individually the following Sections.

#### 3.1.1 Chlorination of the D Unit Backbone of Polymer Donors

##### Chlorination of BDT

**Scheme 1:**
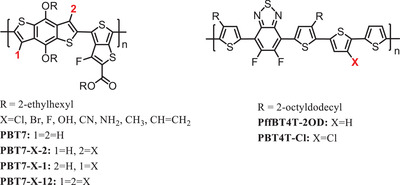
Polymer donors based chlorinated D unit backbone.

Benzo[1,2‐b:4,5‐b′]‐dithiophene (BDT) is one of the most widely used donor units in construction of D–A copolymers for OSCs. In 2009, Yu et al. synthesized a new donor molecule PTB1, incorporating a BDT unit.^[^ ^27^ ^]^ The light absorption range of PTB1 was thus effectively expanded, compared to that of P3HT, which was the main donor material using in OSCs before the arrival of the PTB family of materials. The PTBs optimized the molecular structure, creating a series of molecules, including the star molecule PTB7.^[^ ^28^, ^29^ ^]^ In 2016, Lavarda et al. theoretically studied 24 PTB7 derivatives by selecting different substituents, which were placed at positions 1 or 2 and both 1 and 2 in the PTB7 monomeric units (see **Scheme** **1**).^[^ ^30^ ^]^ The results of evaluation of the electronic and optical properties of these PTB7 derivatives indicated that chlorination produced led to the most promising polymer to apply in OSCs. Among the 3 chlorinated PTB7 derivatives, PTB7‐Cl‐12 was most promising for use in OSCs with PC_61_BM, because it had parameters close to the ideal for *E*_HOMO_, *E*_LUMO,_ and ∆*E*_HL_, which suggested that it would have a higher *V*_OC_ than PTB7.

##### Chlorination of Thiophene

Thiophene (Th) is a weak donor unit. Chlorinated thiophene was used in the benzothiadiazole‐based four‐thiophene polymer and synthesized a new polymer PBT4T‐Cl, by adding a Cl at the fourth position in the central Th as shown in Scheme 1.^[^ ^31^ ^]^ Devices based on PBT4T‐Cl and PC_71_BM have better *V*_OC_ (0.8 V) and fill factor (FF) (74.6%) than the chlorine‐free analogs (the photovoltaic performance is listed in **Table** **1**),^[^ ^31^ ^]^ which is mainly because chlorination on the 4‐position of the middle thiophene lowers the HOMO level of the polymer without causing torsional strain on the backbone. This is helpful to maintain a satisfactory morphology and major face‐on orientation, consequently giving an outstanding PCE of 11.18%. PBT4T‐Cl was calculated to have a larger difference between the ground‐state and excited‐state dipole moments, which suggested less Coulomb binding of excitons. Thus, it is not surprising that PBT4T‐Cl has both elevated hole and electron mobility as shown by the space charge limited current (SCLC) method. Besides, chlorination was found to be effective in stabilizing the photovoltaic performances. PBT4T‐Cl held its high *V*_OC_ in the test and still maintained a PCE of 8.16% after 50 d, which was thought to be because chlorination could steady the two‐phase nanostructure in the active layer to ensure the stability.

**Table 1:** Electronic and photovoltaic performance of polymer donors based chlorinated D unit backbone

Donor	HOMO [eV]	LUMO [eV]	Acceptor	*V*_OC_ [V]	*J*_SC_ [mA cm^−2^]	FF [%]	PCE [%]
PffBT4T‐2OD	−5.19	−3.53	PC_71_BM	0.75	19.23	70.54	10.17
PBT4T‐Cl	−5.33	−3.64	PC_71_BM	0.80	18.71	74.60	11.18

#### 3.1.2 Chlorination of the D Unit Side Chain of the Polymer Donor

Compared to chlorinating the D unit backbone directly, chlorination on the conjugated side chain has attracted more attention. One of the most popular conjugated side chains is thienyl group, which is usually added onto the BDT unit giving BDT‐T, to enhance the coplanarity of the main chain.^[^ ^26^ ^]^ To date, almost all the reported chlorination of the side chain has taken place on the thienyl group of BDT‐T.^[^ ^20^, ^32^, ^33^, ^34^, ^35^, ^36^, ^37^, ^38^ ^]^ As a donor building block of copolymers, BDT has many advantages, including extended conjugation, high planarity, and small steric hindrance. In view of the fact that high planarity is the key feature for the BDT‐T unit, the possibility that the introduction of a chlorine atom with its large atomic radius will break the planarity is a primary concern. Hou et al. calculated the twisting barriers between Th and BDT in BDT‐T‐2F and BDT‐T‐2Cl^[^ ^20^ ^]^ and their results showed that the optimal dihedral angle and twisting barrier of BDT‐T‐2Cl and BDT‐T‐2F were very close. Chlorinated BDT‐T as the donor unit has been used widely together with different acceptor units.

**Scheme 2:**
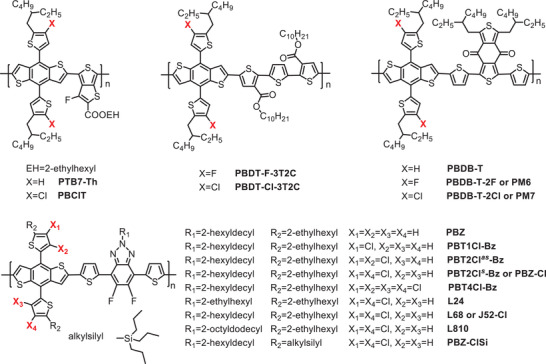
Polymer donors based chlorinated side chains of D units.

In 2018, our group introduced chlorinated BDT‐T into the popular polymer donor, PTB7‐Th, to replace the BDT‐T, and obtained PBClT (see **Scheme** **2**).^[^ ^39^ ^]^ The chlorinated BDT‐T neither increased the steric hindrance of the conjugated backbone or broke the *π*–*π* stacking interactions. Due to the resonance effect of the chlorine atoms, the HOMO level was efficiently decreased and a high *V*_OC_ of > 1 V was achieved in the devices based on PBClT:ITIC (see **Table** **2**). This work showed that the strategy of chlorinating the side chains of polymers could control energy levels well without breaking the intermolecular *π*–*π* stacking interactions.

**Table 2:** Electronic and photovoltaic performance of polymer donors based chlorinated side chain of D unit

Donor	HOMO [eV]	LUMO [eV]	Acceptor	*V*_OC_ [V]	*J*_SC_ [mA cm^−2^]	FF [%]	PCE [%]
PBT1Cl‐Bz	−5.39	−3.57	IT‐4F	0.71	16.85	63.48	7.60
PBT2Cl^as^‐Bz	−5.48	−3.56	IT‐4F	0.79	13.43	41.75	4.43
PBT2Cl^s^‐Bz	−5.47	−3.58	IT‐4F	0.77	15.55	65.21	7.80
PBT4Cl‐Bz	−5.64	−3.57	IT‐4F	0.96	16.42	58.69	9.25
PBZ	−5.18	−3.22	IT‐4F	0.60	17.0	63.2	6.4
PBZ‐Cl	−5.39	−3.41	IT‐4F	0.80	17.7	68.3	9.7
PBZ‐ClSi	−5.56	−4.07	IT‐4F	0.93	19.2	71.5	12.8
PBClT	−5.47	−3.61	ITIC	1.01	13.95	60.05	8.46
J52	−5.13	−3.22	BTA3	1.07	14.62	60.34	9.41
J52‐Cl	−5.39	−3.45	BTA3	1.24	13.16	66.62	10.50
L24	−5.43	−3.48	IT‐4F	0.495	7.41	36.3	1.33
L68	−5.51	−3.56	IT‐4F	0.758	19.50	63.2	9.30
L810	−5.57	−3.58	IT‐4F	0.790	20.76	73.5	12.1
PBDB‐T	−5.34	−3.32	IDT6CN‐M	0.93	16.3	75.9	11.4
PM6	−5.50	−3.61	IDT6CN‐M	1.04	16.6	77.1	13.3
PBDB‐T‐2Cl	−5.52	−3.57	IDT6CN‐M	1.05	16.4	77.5	13.3
PBDT‐F‐3T2C	−5.53	−3.56	IT‐4F	0.852	20.12	68.09	11.67
PBDT‐Cl‐3T2C	−5.56	−3.58	IT‐4F	0.871	18.63	62.80	10.18
PBDB‐T‐2F	−5.50	−3.61	IT‐4F	0.84	20.81	76	13.2
PBDB‐T‐2Cl	−5.52	−3.57	IT‐4F	0.86	21.80	77	14.4

**Figure 1:**
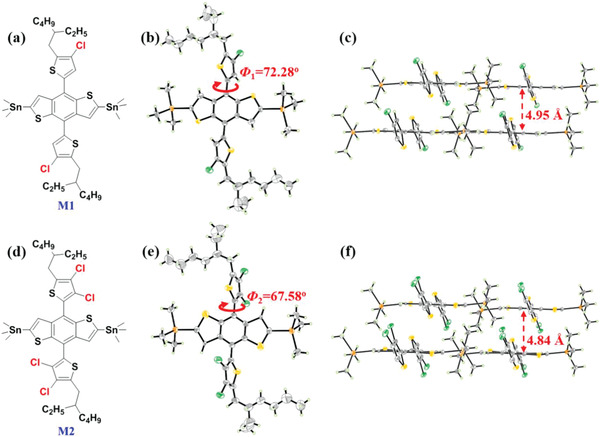
Structure of a) monomer M1 (BDT‐T2Cl^s^) and d) monomer M2 (BDT‐T4Cl), the crystal structure of b) monomer BDT‐T2Cls front view and c) the packing diagrams; e) monomer BDT‐T4Cl front view and f) the packing diagrams. Note: H atoms and 2‐ethylhexyl chains were omitted for clarity. Reproduced with permission.^[^ ^2^ ^]^ Copyright 2019, Elsevier.

BDTT‐alt‐DTBTz is a widely used D–A molecular skeleton, based on which many derivatives with strong optical absorption, ordered molecular packing and high hole mobility have been designed and synthesized by engineering the side chains and/or the halogenation.^[^ ^40^, ^41^, ^42^ ^]^ Several papers about chlorinated the thiophene side chain of this molecular skeleton have been published.^[^ ^32^, ^36^, ^37^, ^38^ ^]^ In this system, our group studied the influence of the position of chlorination and the number of chlorinated sites and found that chlorination had a profound influence on energy levels, light absorbance, molecular dipole moments (*δ*), molecular stacking, morphology control and as a result, photovoltaic performance. We introduced different numbers of chlorine atoms at different sites onto the thienyl‐side chains of BDT‐T and synthesized four polymers with wide bandgaps and deep HOMO levels. These are PBT1Cl‐Bz, PBT2Cl^as^‐Bz, PBT2Cl^s^‐Bz (also named elsewhere PBZ‐Cl, L68 or J52‐Cl) and PBT4Cl‐Bz, whose structures are shown in Scheme 2.^[^ ^32^ ^]^ The four polymers had similar LUMO levels, while the HOMO levels became progressively lower with the increase in the number of chlorine atoms. This could be due to the electron‐withdrawing effect of chlorine and the intermolecular noncovalent interactions of Cl···S and Cl···*π* and led to a hypochromatic shift in the absorption spectra as the number of chlorine atoms increased. We found that PBT2Cl^as^‐Bz and PBT2Cl^s^‐Bz had almost identical LUMO and HOMO levels, indicating the chlorinated sites had little influence on the energy levels in this series of compounds. Their differences were reflected in the twist effects and the solubility at high temperatures. PBT4Cl‐Bz displayed the strongest shoulder peak both in solution and in the solid‐state, implying that it had stronger aggregation than the other three molecules in both states. PBT2Cl^as^‐Bz and PBT4Cl‐Bz, with side chains chloro‐substituted at both the third and fourth positions, had the significant property of temperature‐dependent aggregation (TDA), which enabled morphology control during processing. The simulative *δ* values for the four polymers indicated that the polar effect of Cl was very strong for the entire backbone, and this contributed to the IACT and intermolecular dipole–dipole effect and led to increased *π*–*π* stacking. The PBT4Cl‐Bz:IT‐4F‐based device achieved the highest PCE of 9.25%, with a high *V*_OC_ of 0.96 V while retaining a good *J*_SC_ of 16.04 mA cm^−2^ (see Table 2). A small energy loss, *E*_loss_ = 0.54 eV was also obtained as a result of the deep HOMO level. To study further the influence of chlorination on the geometry and molecular stacking, single crystals of monomers BDT‐T2Cl^s^ with symmetrical two chlorine substituents and BDT‐T4Cl with four chlorine substituents were prepared and analyzed by single crystal X‐ray diffraction (see **Figure** **1**).^[^ ^2^ ^]^ We observed that the dihedral angle between the dichlorinated Th and BDT cores in BDT‐T4Cl was 67.58°, which is ≈5° smaller than that in BDT‐T2Cl^s^. In addition, BDT‐T4Cl exhibits a shorter distance between adjacent faces of BDT planes than that in BDT‐T2Cl^s^, showing that BDT‐T4Cl pack more tightly than BDT‐T2Cl^s^ although with more Cl involved.

**Figure 2:**
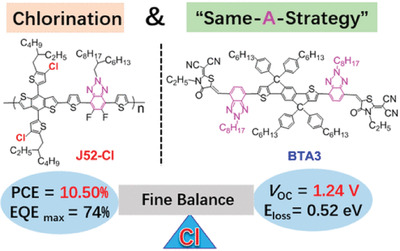
The scheme of chlorination and “Same‐A‐Strategy.” Adapted with permission. ^[^ ^38^ ^]^ Copyright 2019, American Chemical Society.

When the chlorination strategy and side‐chain engineering are combined, the energy level and the morphology can be further controlled. Chen et al. synthesized three polymers based on BDT‐2Cl and a fluorobenzotriazole (FTAZ) unit with different branch alkyl side chains.^[^ ^36^ ^]^ Similar to the work described above, they found the incorporation of chlorine improved the molecular planarity and energy levels, and enhanced the monomer polymerization activity. Upon regulating the side‐chain length of FTAZ unit, they found a large fluctuation of *V*_OC_, *J*_SC,_ and FF, owing to the side chain having a distinct impact on *E*_loss_ and the molecular orientation. Li et al. replaced alkyl groups on the thienyl side chain of BDT‐T by alkylsilyl substituents, and this was seen to be an efficient substitution which deepened the energy levels of BDT‐T,^[^ ^40^ ^]^ and they prepared a new copolymer PBZ‐ClSi (see Scheme 2),^[^ ^37^ ^]^ which showed a reduced HOMO level, enhanced absorption coefficient and increased charge mobility. Subsequently, the PBZ‐ClSi:IT‐4F‐based devices processed by nonhalogenated solvents obtained an outstanding PCE of 12.8% with total improvement of *V*_OC_, *J*_SC_, FF, and a low *E*_loss_ of 0.57 eV. Zhou et al. achieved an excellent *V*_OC_ of 1.24 V in a PSCs device based on J52‐Cl and a non‐fullerene small molecule BTA3 (see **Figure** **2**).^[^ ^38^ ^]^ Interestingly, both the two molecules have the same A unit of benzotriazole, and accordingly they called it “Same‐A‐Strategy” (SAS).

**Figure 3:**
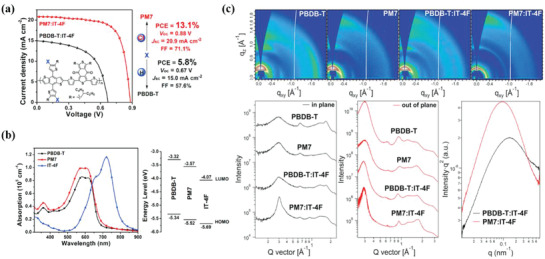
a) Molecular structure and *J–V* curves and b) absorption spectra in solution and energy levels. c) The 2D GIWAXS profiles and the corresponding IP and OOP line‐cuts and the RSoXS scattering profile. Reproduced with permission.^[^ ^34^ ^]^ Copyright 2018, Elsevier.

BDT‐T‐alt‐1,3‐bis(4‐(2‐ethylhexyl)thiophen‐2‐yl)‐5,7‐bis(2‐alkyl)benzo[1,2‐c:4,5‐c′]dithiophene‐4,8‐dione (BDD) is another popular molecular skeleton, based on which many efficient derivatives have been developed.^[^ ^20^, ^33^, ^34^, ^43^, ^44^ ^]^ PBDB‐T‐2Cl, sometimes termed PM7, is one of the efficient derivatives. Hou et al.^20^ and Li et al.^34^ designed a new polymer, PBDB‐T‐2Cl, which consists of a chlorinated‐thienyl benzodithiophene (BDT‐2Cl) donor unit and a benzodithiophene‐4,8‐dione acceptor unit. Compared to PBDB‐T which has no chlorine, PBDB‐T‐2Cl displayed a deeper HOMO, a higher absorption coefficient, increased crystallinity and improved carrier mobility (see **Figure** **3**). In addition, the PBDB‐T‐2Cl:IT‐4F‐based OSCs processed by toluene, a halogen‐free and biodegradable solvent, obtained a high PCE of 13.1% with *V*_OC_ of 0.88 V, *J*_SC_ of 20.9 mA cm^−2^ and FF of 71.1%, while the device based on PBDB‐T:IT‐4F had a poor PCE of 5.8% with low *V*_OC_ of 0.67 V, *J*_SC_ of 15.0 mA cm^−2^ and FF of 57.6%, showing that chlorinated materials have potential in the development of devices with low toxicity.^[^ ^34^ ^]^ Compared with the corresponding fluorinated compound, PBDB‐T‐2Cl is feasible, can be synthesized economically and differs negligibly in calculated molecular configuration, absorption spectra, aggregation, morphology and hole mobility. It holds however, a lower HOMO level which contributes to the higher *V*_OC_.^[^ ^20^ ^]^ When the PBDB‐T‐2Cl:IT‐4F‐based OSCs were processed by chlorobenzene (CB), the PCE was improved to 14.4% (*V*_OC_ = 0.86 V, *J*_SC_ = 21.80 mA cm^−2^ and FF = 77%), which is the highest observed PCE of single‐junction PSC‐based chlorinated materials. In this case, a certified PCE of 13.9% was recorded.^[^ ^20^ ^]^ The study of PBDB‐T reveals that chlorination is a competitive substituent for fluorination in development of high‐performance donor polymers. Zhang et al. also developed efficient OSCs based on PBDB‐T‐2Cl and an asymmetrical nonfullerene acceptor (IDT6CN‐M).^[^ ^33^ ^]^ PBDB‐T‐2Cl:IDT6CN‐M shows quite small HOMO offsets, with ∆*E*_HOMO_ = 0.08 eV because chlorination lowers the HOMO level of PBDB‐T‐2Cl, leading to a high *V*_OC_ of 1.05 V, and a small *E*_loss_ of 0.55 eV. Studies from the Zhang group show that PBDB‐T‐2Cl and IDT6CN‐M have good compatibility of their morphology, and the corresponding device has efficient photon capture and exciton dissociation, and participates in balanced and efficient charge transfer and extraction processes. Consequently, the device based on PBDB‐T‐2Cl:IDT6CN‐M obtained a high PCE of 13.3%. Meanwhile, the system of the fluorinated polymer PM6, and IDT6CN‐M was studied and very similar results were achieved with the PBDB‐T‐2Cl:IDT6CN‐M system.

Chlorinated BDT‐T donor units were also reported to copolymerize with thiophene (T) to synthesize a D–A‐type copolymer PBDT‐Cl‐3T2C.^[^ ^35^ ^]^ The corresponding fluorinated counterpart, PBDT‐F‐3T2C was also synthesized by Chen et al. and both the structures are shown in Scheme 2. The effect of chlorination on energy levels is similar to that in BDT‐T based on other copolymers and other A units mentioned previously. A chlorine atom possesses a stronger ability than a fluorine atom to deepen energy levels. However, a fluorine atom with its smaller size possesses a stronger self‐assembly ability and minimal steric hindrance, and leads to PBDT‐F‐3T2C with higher crystallinity and TDA behavior in solution than PBDT‐Cl‐3T2C. Finally, the fluorinated polymer PBDT‐F‐3T2C with a higher absorption coefficient, better morphology, higher and more balanced charge mobilities shows a higher PCE than PBDT‐Cl‐3T2C.

From these cases, it can be seen that chlorination and fluorination have some similar characteristics and some unique features. The results of comparing the final photovoltaic performance of fluorinated and chlorinated copolymers show that they are different on a case by case basis. Consequently, we can select a proper strategy consistent with different situations.

#### 3.1.3 Chlorination of the A Unit of a Polymer Donor

**Scheme 3:**
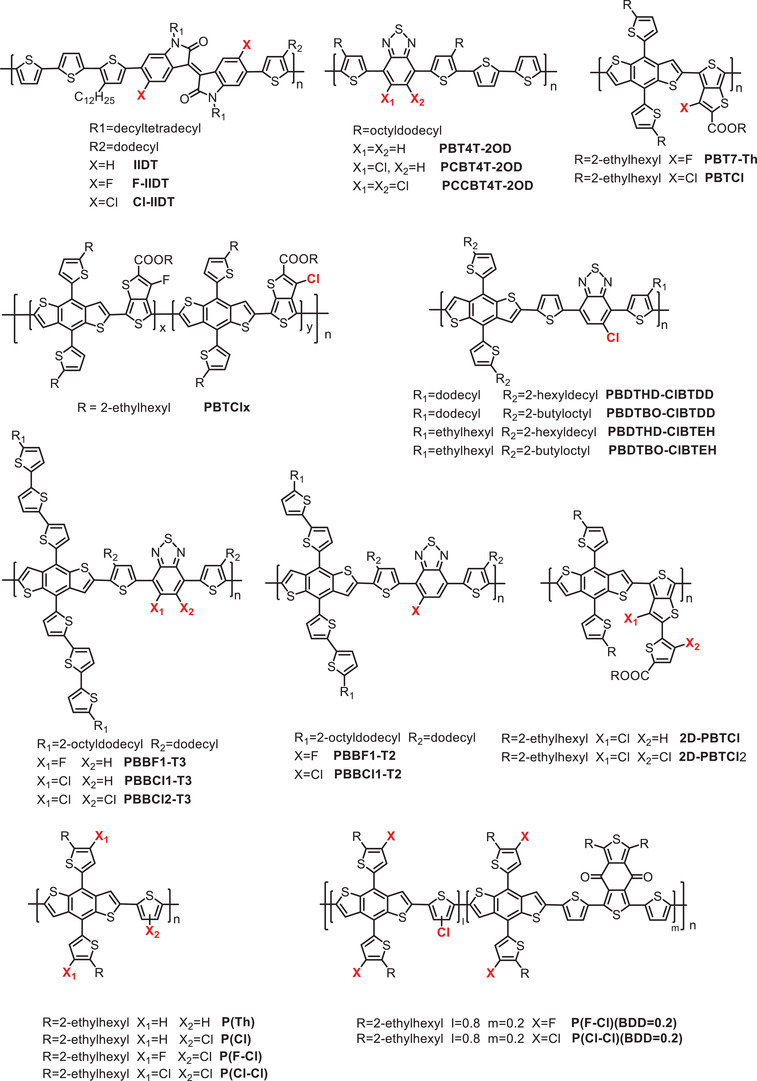
Polymer donors based chlorinated backbone of A units.

So far, the A units that are chlorinated, are mainly isoindigo (IIDT),^[^ ^23^ ^]^ thienothiophene (TT),^[^ ^45^, ^46^, ^47^ ^]^ and benzothiadiazole (BT).^[^ ^48^, ^49^, ^50^ ^]^ In this section, we will introduce each of them. The structures and the electronic and photovoltaic performance of the related molecules are shown in **Scheme** **3** and **Table** **3**, respectively.

**Table 3:** Electronic and photovoltaic performance of polymer donors based chlorinated backbone of A unit

Donor	HOMO [eV]	LUMO [eV]	Acceptor	*V*_OC_ [V]	*J*_SC_ [mA cm^−2^]	FF [%]	PCE [%]
F‐IIDT	−5.51	−3.92	PC_71_BM	0.63	4.26	44	1.19
Cl‐IIDT	−5.53	−3.85	PC_71_BM	0.75	10.00	61	4.60
PBT4T‐2OD	−5.12	−3.63	PC_71_BM	0.70	10.58	65.45	4.89
PCBT4T‐2OD	−5.26	−3.59	PC_71_BM	0.73	16.18	68.97	8.21
PCCBT4T‐2OD	−5.32	−3.62	PC_71_BM	0.85	11.93	60.14	6.12
PBTCl	−5.73	−3.69	PC_71_BM	0.92	10.24	50.23	4.75
PBTCl75	−5.50	−3.70	PC_71_BM	0.89	11.55	57.86	5.90
PBTCl50	−5.36	−3.71	PC_71_BM	0.85	13.02	61.03	6.79
PBTCl25	−5.36	−3.71	PC_71_BM	0.82	15.31	66.19	8.31
PBTCl20	−5.35	−3.72	PC_71_BM	0.81	13.94	66.00	7.47
PBTCl10	−5.35	−3.73	PC_71_BM	0.80	14.07	69.28	7.79
PBT7‐Th	−5.29	−3.72	PC_71_BM	0.79	14.98	66.44	7.91
PBT7‐Th	−5.29	−5.43	ITIC	0.80	14.34	58	6.62
PBTCl	−3.72	−3.55	ITIC	0.91	14.53	58	7.57
PBT7‐Th	−5.38	−3.70	ITIC	0.82	14.76	57.09	6.91
2D‐PBTCl	−5.45	−3.60	ITIC	0.91	15.79	61.32	8.81
2D‐PBTCl2	−5.43	−3.63	ITIC	0.87	14.52	58.43	7.38
PBDTHD‐ClBTDD	−5.53	−3.71	PC_71_BM	0.76	16.79	71.69	9.11
PBDTBO‐ClBTDD	−5.47	−3.70	PC_71_BM	0.68	11.69	62.30	4.95
PBDTHD‐ClBTEH	−5.53	−3.71	PC_71_BM	0.79	13.33	63.12	6.88
PBDTBO‐ClBTEH	−5.50	−3.73	PC_71_BM	0.78	10.94	64.36	5.46
PBBF1‐T2	−5.44	−3.20	PC_71_BM	0.81	9.48	45.37	3.49
PBBCl1‐T2	−5.49	−3.22	PC_71_BM	0.87	8.44	49.49	3.64
PBBF1‐T3	−5.45	−3.21	PC_71_BM	0.73	12.74	66.94	6.21
PBBCl1‐T3	−5.44	−3.38	PC_71_BM	0.73	13.75	68.59	6.87
PBBCl2‐T3	−5.50	−3.21	PC_71_BM	0.84	9.90	63.94	5.33
P(Th)	−5.32	−3.32	ITIC‐Th	0.779	8.8	37.3	2.6
P(Cl)	−5.47	−3.50	ITIC‐Th	0.905	20.63	65.0	12.14
P(F‐Cl)	−5.64	−3.66	IT‐4F	0.879	20.3	66.0	11.8
P(Cl‐Cl)	−5.70	−3.68	IT‐4F	0.899	20.3	56.1	10.2
P(F‐Cl)(BDD = 0.2)	−5.62	−3.74	IT‐4F	0.838	21.3	71.0	12.7
P(Cl‐Cl)(BDD = 0.2)	−5.67	−3.77	IT‐4F	0.899	21.7	67.5	13.2

##### Chlorination of IIDT

In 2013, polymers containing chlorinated IIDT were found to provide a significant increase in electron mobility in organic field effect transistor (OFET) devices, showing that chlorination has the potential to tune the electronic properties of the conjugated polymers.^[^ ^51^ ^]^ Subsequently, Zheng et al. introduced chlorinated isoindigo, Cl‐IIDT, into a polymer,^[^ ^23^ ^]^ and found that in addition to tuning the frontier energy levels, chlorination could increase the torsion angle of the polymer backbone. The computational analysis showed that the dihedral angle of F‐IIDT main chain was ≈11°, and the distance between the F and H atoms (2.27 Å) was shorter than the sum of their van der Waals radii (2.56 Å), suggesting strong interactions between F and H. This leads to large crystal domains in blended films with PC_71_BM and a low PCE of 1.19%. In contrast, a chlorine atom with a much larger atomic radius made the Cl‐IIDT skeleton less planar with the phenyl‐thienyl torsional angle as high as 40°. Compared to the corresponding fluorinated compound, Cl‐IIDT has a small but suitable size of crystallized domains and a large “face‐on” conformation, which favors charge transfer. Due to the large atomic size of chlorine and the weaker molecular interactions, Cl‐IIDT could intermingle with PC_71_BM sufficiently in solution and precipitate slowly to form a favorable morphology in film processing, resulting in better photovoltaic performances with PCE = 4.60%.

##### Chlorination of TT

In 2017, to utilize the power of chlorination more effectively, our group synthesized an array of terpolymers, PBTClx, and optimized their photovoltaic performance by adjusting the ratio of fluorinated TT (TT‐F) and chlorinated TT (TT‐Cl) monomers.^[^ ^46^ ^]^ As the portion of TT‐Cl increases, the twist angle between the donor unit and the acceptor unit increased, resulting in a drop in the planarity and conjugation. Consequently, the absorption spectrum was continuously hypochromatically shifted, and the bandgap gradually increased from 1.57 to 2.04 eV as the chlorinated units were increased from 0% to 100%. The HOMO levels of the polymers were deepened by enhancing the amount of chlorinated units, and this offered an approach to tune the *V*_OC_ continuously, eventually achieving a balance and an optimized performance. The best PCE of 8.31% was achieved when adopting PBTCl_25_ together with PC_71_BM in the device, and in this instance, the *V*_OC_ was enhanced from 0.79 to 0.82 V. From the morphology study, torsion and aggregation of blends could be fine‐tuned with careful optimization of the TT‐Cl ratios to favor charge transfer. This study showed that introducing chlorinated moieties to terpolymers could be a useful tool toward generation of better PCE.

Since chlorination of thieno[3,b]thiophene would cause a blue‐shift of polymers in the UV–vis absorption spectra compared to the fluorinated counterpart, we fabricated devices using PBTCl as the donor and ITIC as the acceptor.^[^ ^47^ ^]^ For the more complementary light absorbance of PBTCl and ITIC, devices based on these species gave a PCE of 7.57%, registering a 13% increase compared to the fluorinated material. Films of the blends of PBTCl and ITIC showed a “face‐on” conformation and good phase separation, promoting charge transfer and a high PCE.

To alter the torsion and steric hindrance caused by the chlorination of thieno[3,b]thiophene, the design strategy of the 2D side chain was applied^[^ ^45^ ^]^ with the purpose of increasing the molecular interactions and the *π*–*π* stacking. Compared to the unsubstituted 1D analog, 2D‐PBTCl still has a blue‐shifted absorption to match better with ITIC and a deeper HOMO level to give higher *V*_OC_. Devices with ITIC and 2D‐PBTC showed a PCE of 8.67%, which is higher than that of PTB7‐Th and ITIC‐based devices. This indicates that the strategy of 2D side chain obtained from extending conjugation behaves synergistically with chlorination to reduce the steric effect and improve the photovoltaic performance.

##### Chlorination of BT

In 2016, our group monochlorinated the BT moiety to obtain a series of asymmetric polymers, which were used to study the influence of chlorination on photovoltaic performance.^[^ ^49^ ^]^ It was found that chlorination of the BT unit deepened the HOMO levels and increased the *V*_OC_ of devices blended with PC_71_BM as the active layer, resulting in a high PCE of up to 9.11%. Though chlorination would cause large torsion on the polymer chain due to the large atomic size of chlorine, a favorable backbone orientation is still maintained for efficient photovoltaic applications.

In 2017, we designed and synthesized a type of conjugated polymer based on chlorinated BT moiety, and produced monochlorinated PCBT4T‐2OD and dichlorinated PCCBT4T‐2OD.^[^ ^48^ ^]^ A significant increase from 4.89% to 8.21% of the PCE was observed when comparing PCBT4T‐2OD with the nonchlorinated PBT4T‐2OD. This increase stems from improvements in *V*_OC_, *J*_SC,_ and FF. It was found that *V*_OC_ of photovoltaic devices increased as the number of chlorine atoms increases, which is consistent with the observation in cyclic voltammograms that HOMO levels deepen in the order of PBT4T‐2OD, PCBT4T‐2OD, and PCCBT4T‐2OD. Chlorinated substitution on the BT moiety would enhance *J*_SC_, but monochlorination does much better than dichlorination. This is probably because of the better charge transfer in the blends which is confirmed by SCLC measurements of the hole mobility of PCBT4T‐2OD which is much higher than that of PBT4T‐2OD and PCCBT4T‐2OD. Further, GIWAXS shows that PCBT4T‐2OD has a better mixture of both “edge‐on” and “face‐on” conformations and a better crystalline condition than PBT4T‐2OD or PCCBT4T‐2OD, which would favor charge transport to give improved photovoltaic performance. The polymer backbone in PCCBT4T‐2OD is contorted severely, making the molecular interaction too weak to form an efficient morphology. This study concluded reasonably, that chlorinated substitution would modify the bandgaps and alter the morphology and orientation of polymers towards higher PCE values. Later, our group continued to introduce fluorine atoms, obtaining PCFBT4T‐2OD, which shows a further improvement with PCE = 8.84% and especially = *J*_SC_ = 17.61 mA cm^−2^.^[^ ^52^ ^]^ the external quantum efficiency (EQE) of PCFBT4T‐2OD has relatively high values from 450 to 700 nm. Comparing PCFBT4T‐2OD and PCBT4T‐2OD, the grazing incidence wide‐angle X‐ray scattering (GIWAXS) showed a higher ratio of “face‐on” conformation under the combined effect of chlorine and fluorine atoms, which benefits vertical charge transfer between electrodes. Consequently, chlorinated substitution can enhance *V*_OC_ and fluorinated substitution would improve *J*_SC_ generally, which means that strategic use of chlorination in materials would be a useful way to develop OSCs with excellent photovoltaic performance.

In 2018, we used a chlorinated BT unit to develop 2D PSCs.^[^ ^50^ ^]^ Such chlorinated polymers have broader absorption and yield high *J*_SC_ and deep HOMO levels to give a high *V*_OC_ compared to the fluorinated units, among which PBBCl1‐T3 has the highest PCE of 6.87% when blended with PC_71_BM. Additionally, the advantages of chlorination over fluorination would be hindered because the *π*‐conjugated side chains lengthen as the side chains reduce the torsion of polymer chains caused by the chlorine atom. Although devices based on dichlorinated PBBCl2‐T3 have the highest *V*_OC_ of 0.87 V, they do not have a satisfactory *J*_SC_ probably because chlorine atoms disrupt the polymer backbone planarity and the molecular interactions.

##### Chlorination of Th

**Figure 4:**
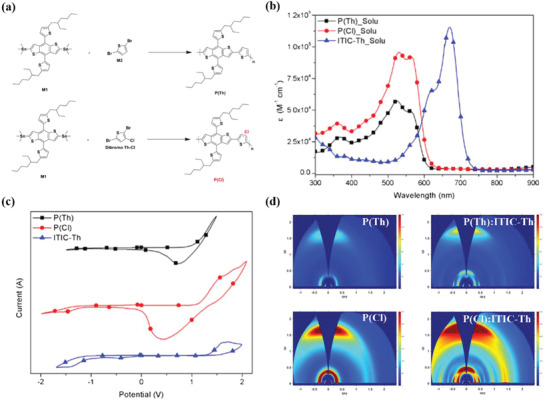
a) Synthesis routes, b) molar absorption coefficients, c) CV curves for the CF thin films, d) 2D GIWAXS data. Reproduced with permission.^[^ ^54^ ^]^ Copyright 2019, Wiley.

Thiophene can be used as a weak donor unit, but when it is combined with a stronger donor unit, it also can play the role of the acceptor unit. Chlorinated thiophene (Cl‐Th) behaves similarly.^[^ ^53^, ^54^, ^55^ ^]^ Recently, with Cl‐Th as the acceptor unit, Moon et al. synthesized a D–A copolymer, P(Cl) (see Scheme 3), ^[^ ^54^ ^]^ with low synthetic complexity, high yield, and low cost. Their calculations found that P(Th), the control molecule without any Cl substituent, had a twisted and linear curvature, while P(Cl) had a more twisted and zig‐zagged curvature due to the large atomic radius of Cl. In addition, chlorination led to a higher molecular weight, an outstanding absorption coefficient, red‐shifted absorption, a deeper HOMO and stronger *π*–*π* stacking as shown in **Figure** **4**. It is interesting that when blended with ITIC‐Th, the films showed even higher crystallinity and smaller *π*–*π* stacking distance and the resulting device showed an excellent certified PCE of 12.14% and outstanding stability. They further fluorinated and chlorinated the Th side chains of the BDT unit fabricating P(F–Cl) and P(Cl–Cl) (see Scheme 3), respectively.^[^ ^55^ ^]^ As a result, both the HOMO levels decreased but that of P(Cl–Cl) decreased more. Further fluorination and chlorination led to excessive aggregation and thus low solubility and consequently lower molecular weight. In order to decrease the aggregation, they incorporated a small proportion of the BDD unit and this led to enhanced molecular weights and solubilities. The solubilities of the new polymers in eco‐friendly o‐xylene were also very good, which was an added advantage of this system. The devices based on P(F–Cl)(BDD = 0.2):IT‐4F and P(Cl–Cl)(BDD = 0.2):IT‐4F processed by o‐xylene gave PN (99.5:0.5, v/v%) and obtained excellent certified PCEs of 12.70% and 13.97%, respectively.

#### 3.1.4 Chlorination of the *π*‐Bridge of the Polymer Donor

The D–A‐type structure has been widely applied in the design of efficient polymers. The strong IACT between D unit and A unit can lead to a narrow energy gap and also expand the absorption. As the research further develops, derivation takes the place of a *π*‐bridge, which is a key linkage between the D and A units and can modulate the skeletons of conjugated polymers. The *π*‐bridge plays a crucial role in tuning the light absorption, the bandgap, and energy levels of D‐*π*–A‐*π* type conjugated materials. To date, Th is one of the most classical *π*‐bridges, which has constructed conjugated polymers.

**Scheme 4:**
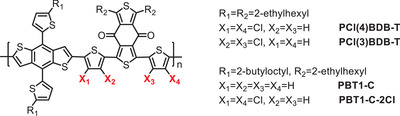
Polymer donors based chlorinated *π*‐bridges.

Recently, Sun et al. synthesized a new polymer, PBT1‐C‐2Cl, by chlorinating the thiophene *π*‐bridge of PBT1‐C (see **Scheme** **4**).^[^ ^56^ ^]^ To better study the influence of chlorination, density functional theory (DFT) calculations at the B3LYP/6‐31 G (d, p) level were performed. Owing to the larger size of the Cl atom than that of H, the dihedral angle between BDT and chlorinated Th was increased. The twisted configuration and the high electronegativity of Cl both play an important part in reducing the HOMO of PBT1‐C‐2Cl, which was confirmed by cyclic voltammetry (CV) measurements and contributes to the improvement of *V*_OC_. The chlorination of the thiophene *π*‐bridge can also tune the electron cloud distributions. It was observed that the electron cloud of PBT1‐C‐2Cl is more delocalized, with a higher electron cloud density in the BDD units and a lower density in the BDT units from the HOMO level to the LUMO level. The PBT1‐C‐2Cl:IT‐4F‐based device achieved a somewhat higher PCE of 12.7% compared to PBT1‐C:IT‐4F‐based device (PCE = 10.9%), due to the enhancement of *V*_OC_ and FF (see **Table** **4**). The latter is supposed to be attributed to the film morphology measured by AFM and TEM and the enhancement of the hole mobility. Two different *π*–*π* stacking distances were found for both blend films and although the chlorine atom is larger than the hydrogen atom, there was a larger proportion of tight packing regions in the PBT1‐C‐2Cl:IT‐4F film, which can explain the high hole mobility shown by the SCLC data. The introduction of chlorine can also decrease the miscibility with IT‐4F and enhance domain purity, leading to reduced charge recombination.

**Table 4:** Electronic and photovoltaic performance of polymer donors based chlorinated *π*‐bridge

Donor	HOMO [eV]	LUMO [eV]	Acceptor	*V*_OC_ [V]	*J*_SC_ [mA cm^−2^]	FF [%]	PCE [%]
PCl(3)BDB‐T	−5.54	−3.27	IT‐4F	0.88	0.79	24.47	0.16
PCl(4)BDB‐T	−5.48	−3.47	IT‐4F	0.84	20.14	71.33	12.11
PBT1‐C	−5.47	−3.46	IT‐4F	0.79	19.70	70.5	10.9
PBT1‐C‐2Cl	−5.59	−3.53	IT‐4F	0.85	19.74	76.0	12.7

Hou et al. studied the effect of the orientation of chlorinated thiophene on D‐*π*–A‐*π* type conjugated polymers.^[^ ^57^ ^]^ They investigated two chlorinated conjugated materials, PCl(3)BDB‐T (P3) and PCl(4)BDB‐T (P4), in which the Th bearing chlorine faced the BDD or the BDT unit, respectively. The small change in orientation caused a huge difference in light absorption, molecular aggregation, charge mobility, morphology and finally photovoltaic performance. They found that in dilute CB solution, P3 showed a maximum absorption peak (*λ*_max_) at 465 nm, while P4 exhibited a *λ*_max_ at 647 nm at room temperature (RT), indicating that P4 had more effective electronic delocalization than the P3 molecule. P3 had a quite weak aggregation, while P4 had a stronger aggregation, which was indicated by the temperature‐dependent light absorption experiments. When the solutions were cooled from 120 to 20 °C, the *λ*_max_ of both molecules exhibited a redshift of about 6 nm and about 100 nm for P3 and P4, respectively. This difference was ascribed to their different twisted conjugated backbones, and this was confirmed by DFT calculations. They carried out DFT calculations and found that P3 had a highly twisted conjugated backbone and the electron in the HOMO level of P4 was not delocalized on BDD units, which limited the IACT. In stark contrast, electrons in HOMO of P4 were well delocalized upon the conjugated backbone. The charge carrier mobilities of both polymer‐based pure and blend films were evaluated and it was found that the pure P3 and P3:IT‐4F blend films did not display any charge transport characteristics after several measurements, while the pure P4 film showed a hole mobility of 2.57 × 10^−5^ cm^2^ V^−1^ s^−1^, and the P4:IT‐4F blend film exhibited a balanced hole and electron mobility of 5.74 × 10^−6^ and 5.16 × 10^−6^ cm^2^ V^−1^ s^−1^, respectively. In addition, the P3:IT‐4F blend film had a less ordered and poor phase‐separation morphology, which was confirmed by the out‐of‐plane X‐ray diffraction (XRD), the AFM and limited the intermolecular charge transfer (IECT). When the polymers were combined with IT‐4F to fabricate OSCs, the P4:IT—4F‐based device achieved a quite high PCE of 12.33%, while the P3:IT‐4F‐based device showed an extremely low PCE of 0.18%, which was ascribed to the very poor IACT and IECT in the P3:IT‐4F film due to the highly twisted conjugated skeleton of P3 and poor morphology of P3:IT‐4F blend film. In contrast, the device‐based P4:IT‐4F film performed well, due to the planar conjugated skeleton of the P4 and a well‐balanced hole and electron transport and the proper phase separation morphology of P4:IT‐4F blend film.

### 3.2 Progress of Chlorinated Small Molecular Acceptors

Small molecule nonfullerene acceptors (NFA) with strong and wide ranging absorption are drawing increasing attention. Recently, OSCs based on small molecule NFAs are developing very rapidly and the PCEs of single‐junction devices have been observed to be over 16% due to the structural optimization of small molecule NFAs. Halogenation is a frequently used strategy in highly efficient systems, especially chlorination and fluorination. When chlorination is used on a small molecular acceptor, some properties are kept the same as those of chlorinated polymer donors. For example, the HOMO levels were all deepened to different degrees. However, some properties such as the shift of light absorption were totally different. In this section, we will describe the progress of chlorinated small molecular acceptors and the specific influence of chlorination on small molecular acceptors.

#### 3.2.1 Chlorination of IC

The IC (2‐(3‐oxo‐2,3‐dihydroinden‐1‐ylidene) malononitrile) unit is an effective electron‐withdrawing group and is one of the most often used end group of low bandgap small molecule acceptors, in particular efficient small molecule acceptors that have been developed recently. The chlorination of IC was found to be a very effective way to enhance its electron‐withdrawing ability. Small molecules with chlorinated IC as an end group show an ultralow bandgap and a near‐infrared absorption spectrum.

##### Monochlorination

**Scheme 5:**
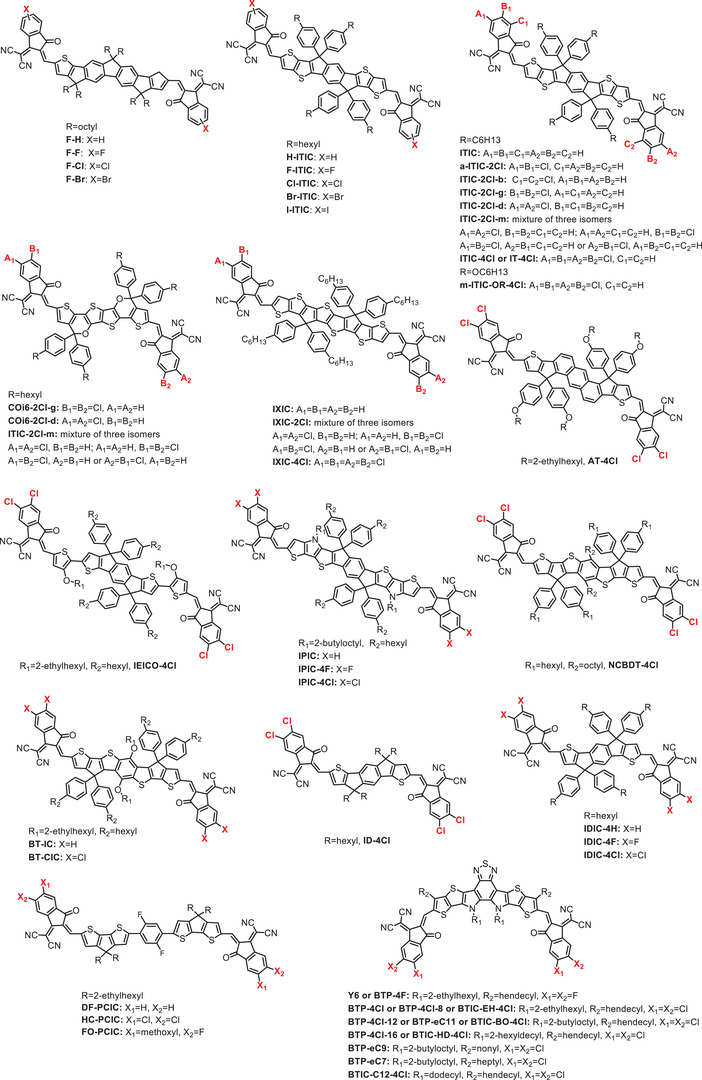
Small molecule acceptors based chlorinated IC end groups.

In 2017, Li et al. developed an array of small molecules with or without halogens (F, Cl, Br, and I) but with the same conjugated core, denoted as ITIC (3,9‐bis(2‐methylene‐(3‐(1,1dicyanomethylene)‐indanone))‐5,5,11,11‐tetrakis(4‐hexylphenyl)di‐thieno[2,3‐d:20,30‐d0]‐s‐indaceno[1,2‐b:5,6‐b0]dithiophene) (see **Scheme** **5**), for use in OFETs and OSCs.^[^ ^58^ ^]^ These are all monohalogenated small molecules with absorption range from 500 to 750 nm in CHCl_3_, and the absorption peak is bathochromic‐shifted in order from H— to F—, Cl—, Br— and I‐ITIC due to the inductive effect and *π*‐electrons delocalizing into empty d‐orbitals of halogens. They found that both the HOMO and LUMO levels of F—, Cl—, and Br‐ITIC are gradually shifted to a deeper position, while I‐ITIC shows very small higher energy levels than that of Br‐ITIC. In addition, the halogenated ITICs show better crystallinity. Devices were fabricated based on these halogenated ITIC derivatives as the nonfullerene electron acceptor and a wide bandgap polymer, PTPDBDT, as the donor. The electron mobilities, except that of F‐ITIC, of halogenated molecules were improved. The PCEs of devices based on all of the halogenated ITIC derivatives were improved, compared to the device based‐H‐ITIC due to the enhanced *J*_SC_ and FF, although the *V*_OC_s dropped from 1.04 V to 0.93–0.95 V (see **Table** **5**). Among all of the devices, the device base‐Cl‐ITIC received the highest PCE of 9.5%. In 2018, Chen et al. also showed the effect of different halogenation on another molecule. They synthesized F—F, F—Cl, F—Br compounds by halogenating the end group of 2,9‐bis(2‐methylene(3‐(1,1‐dicyanomethylene)‐indanone))‐7,12‐dihydro‐4,4,7,7,12,12‐hexaoctyl‐4H‐cyclopenta [2″,1″:5,6;3″,4″:5′,6′]diindeno[1,2‐b:1′,2′‐b′]dithiophene (FDICTF, F—H).^[^ ^59^ ^]^ It was also discovered that after halogenation of the end groups, the NFA molecules showed stronger and bathochromic‐shifted absorption, increased crystallinity and higher charge carrier mobility. However, the influence of halogenation on energy levels is different from that on ITIC. The fluorination deepened the energy levels more efficiently than chlorination and bromination. By combining with PBDB‐T, the F—H, F—F—, F—Cl—, and F—Br‐based optimized devices achieved PCEs of 9.59%, 10.85%, 11.47%, and 12.05%, respectively.

**Table 5:** Electronic and photovoltaic performance of Small molecule acceptors based chlorinated IC end groups

Acceptor	HOMO [eV]	LUMO [eV]	Donor	*V*_OC_ [V]	*J*_SC_ [mA cm^−2^]	FF [%]	PCE [%]
F–H	−5.42	−3.79	PBDB‐T	0.94	15.02	67	9.59
F–F	−5.72	−4.13	PBDB‐T	0.88	17.36	71	10.85
F–Cl	−5.62	−4.04	PBDB‐T	0.87	17.61	75	11.47
F–Br	−5.62	−4.06	PBDB‐T	0.87	18.22	76	12.05
H‐ITIC	−5.61	−4.02	PTPDBDT	1.04	10.6	58	6.4
F‐ITIC	−5.65	−4.09	PTPDBDT	0.94	14.1	66	8.8
Cl‐ITIC	−5.70	−4.14	PTPDBDT	0.94	15.6	65	9.5
Br‐ITIC	−5.73	−4.20	PTPDBDT	0.93	15.4	66	9.4
I‐ITIC	−5.68	−4.14	PTPDBDT	0.95	14.5	65	8.9
a‐ITIC‐2Cl	−5.29	−3.77	PBDB‐T2F	0.88	18.91	73.50	12.23
ITIC‐2Cl‐*β*	−5.30	−3.71	PBDB‐T2F	0.94	18.47	64.63	11.21
ITIC‐2Cl‐*γ*	−5.55	−3.92	PBDB‐T2F	0.93	18.94	74.07	13.03
ITIC‐2Cl‐*δ*	−5.53	−3.94	PBDB‐T2F	0.90	18.34	69.87	11.51
ITIC‐2Cl‐m	−5.54	−3.93	PBDB‐T2F	0.90	18.03	66.86	10.85
IT‐2Cl	−5.68	−3.99	PBDB‐T2F	0.922	19.08	74.8	13.16
IT‐4Cl	−5.75	−4.09	PBDB‐T2F	0.790	22.67	75.2	13.45
IT‐4Cl	−5.75	−4.06	PBFTT	0.76	19.7	73.9	11.1
m‐ITIC‐OR‐4Cl	−5.78	−4.05	BO2FC8	0.83	21.2	63.3	11.0
COi6‐2Cl‐*γ*	−5.46	−4.05	PTB7‐Th	0.69	20.33	62.84	8.82
COi6‐2Cl‐*δ*	−5.46	−4.02	PTB7‐Th	0.67	20.65	60.81	8.41
COi6‐2Cl‐m	−5.47	−4.02	PTB7‐Th	0.69	20.78	64.32	9.22
IXIC	−5.13	−3.78	PBDB‐T	0.82	20.9	65.4	11.3
IXIC‐2Cl	−5.20	−3.90	PBDB‐T	0.73	23.6	70.9	12.2
IXIC‐4Cl	−5.20	−3.95	PBDB‐T	0.69	22.9	71.2	11.2
AT‐NC	−5.44	−3.80	PBDB‐T	0.919	17.11	69.4	10.91
AT‐4Cl	−5.71	−3.89	PBDB‐TF	0.901	19.52	75.5	13.27
IEICO‐4Cl	−5.56	−4.23	J52	0.700	23.8	60.7	10.1
IEICO‐4Cl			PBDB‐T	0.744	20.8	62.5	9.67
IEICO‐4Cl			PTB7‐Th	0.727	22.8	62.0	10.3
IPIC	−5.47	−3.82	PBDB‐T	0.950	7.16	58.6	3.98
IPIC‐4F	−5.54	−3.94	PBDB‐T	0.835	19.8	67.1	11.1
IPIC‐4Cl	−5.51	−3.95	PBDB‐T	0.813	22.2	74.0	13.4
NCBDT‐4Cl	−5.60	−4.02	PBDB‐T‐SF	0.851	22.35	74.3	14.1
BT‐IC	−5.32	−3.85	PTB7‐Th	0.81	17.5	59.6	8.3
BT‐CIC	−5.49	−4.08	PTB7‐Th	0.70	22.5	71.0	11.2
ID‐4Cl	−5.81	−3.97	PM6	0.767	17.87	74.76	10.25
IDIC‐4H	−5.80	−3.87	PBDB‐T2Cl	0.98	10.32	45.30	4.57
IDIC‐4F	−5.95	−3.93	PBDB‐T2Cl	0.88	13.00	61.91	7.10
IDIC‐4Cl	−6.03	−4.02	PBDB‐T2Cl	0.83	16.21	68.69	9.24
DF‐PCIC	−5.49	−3.77	PTQ10	1.04	6.51	54.13	3.68
DF‐PCIC	−5.49	−3.77	PBDB‐T	0.89	15.28	61.87	8.43
FO‐PCIC	−5.49	−3.75	PBDB‐T	0.90	15.02	61.12	8.32
HC‐PCIC	−5.54	−3.87	PTQ10	0.94	15.99	67.96	10.42
HC‐PCIC	−5.54	−3.87	PBDB‐T	0.73	17.53	69.08	9.03
HC‐PCIC	−5.54	−3.87	PBDB‐TF	0.89	18.13	72.06	11.75
Y6	−5.65	−4.10	PBDB‐TF	0.83	25.3	74.8	15.7
BTP‐4Cl	−5.68	−4.12	PBDB‐TF	0.867	25.4	75.0	16.5
BTP‐4Cl‐8			PBDB‐TF	0.872	25.2	74.3	16.3
BTP‐4Cl‐12			PBDB‐TF	0.858	25.6	77.6	17.0
BTP‐4Cl‐16			PBDB‐TF	0.862	24.2	74.8	15.6
BTIC‐C12‐4Cl	−5.56	−4.16	PBDB‐TF	0.84	19.82	68.27	11.36
BTIC‐EH‐4Cl	−5.58	−4.19	PBDB‐TF	0.83	25.13	68.64	14.28
BTIC‐BO‐4Cl	−5.54	−4.14	PBDB‐TF	0.85	25.26	76.25	16.43
BTIC‐HD‐4Cl	−5.58	−4.14	PBDB‐TF	0.86	23.24	69.781	13.95
BTP‐eC7			PBDB‐TF	0.843	24.1	73.5	14.9
BTP‐eC9			PBDB‐TF	0.839	26.2	81.1	17.8
BTP‐eC11			PBDB‐TF	0.851	25.7	77.5	16.9

So far, most NFAs with a monochlorinated IC end group are a mixture of two kinds of isomers due to the lack of site specificity of chlorine.^[^ ^58^, ^59^ ^]^ To study the role of the position of Cl on the end group, we tried to separate the mixed monomers of IC‐Cl‐*δ* and IC‐Cl‐*γ* by recrystallization with selected solvents and successfully isolated ITIC‐2Cl‐*δ* and ITIC‐2Cl‐*γ*. ITIC‐2Cl‐m was also synthesized for comparison. The precise chlorine atom positioning provided us an opportunity to cultivate single crystals and study the properties and the influence of individual isomers on the photovoltaic performance. Compared to ITIC‐2Cl‐*δ*, ITIC‐2Cl‐*γ* exhibited a 0.02 eV higher LUMO and 0.02 eV lower HOMO, resulting in a wider bandgap and a slight blueshift of the absorption spectrum. Both the LUMO and HOMO levels of ITIC‐2Cl‐m are between that of ITIC‐2Cl‐*δ* and ITIC‐2Cl‐*γ*. The higher LUMO level of ITIC‐2Cl‐*γ* would result in a higher *V*_OC_ in devices. The precise Cl position at the end groups of ITIC‐2Cl has a deep influence on the molecular stacking. The single‐crystal structure showed that ITIC‐2Cl‐*γ* had better planarity and a shorter *π*–*π* interaction distance. Moreover, ITIC‐2Cl‐*γ* formed a 3D rectangle‐like interpenetrating network, which was advantageous for charge transport in multiple directions, while ITIC‐2Cl‐*δ* formed only a linear stacked structure. As a result, the device based ITIC‐2Cl‐*γ* with PBDB‐T2F as donor achieved the best PCE of 13.03% due to the all‐round improvement of *V*_OC_, FF, and *J*_SC_ compared to the device‐based ITIC‐2Cl‐*δ*:PBDB‐T2F and ITIC‐2Cl‐m:PBDB‐T2F. Moreover, the best stability was achieved in devices based on ITIC‐2Cl‐*γ*. This revealed that the position of Cl played a key role in the acceptors; moreover, the 3D network structure might be a promising strategy to design better NFAs to enhance the PCE and stability of OSCs. In another project, our group synthesized the third isomers of ITIC‐2Cl‐*β*,^[^ ^60^ ^]^ with a linear packing structure and a higher *V*_OC_ of 0.94 V in the device‐based and with PBDB‐T2F as the donor. However, the influence of the precise Cl position at the IC end groups on the device performance is different in various systems. Recently, we designed and synthesized three new chlorinated acceptors, COi6‐2Cl‐*γ*, COi6‐2Cl‐*δ*, and COi6‐2Cl‐m based on an electron‐rich core, COi6.^[^ ^61^ ^]^ In this work, the device based‐COi6‐2Cl‐*γ* and with PTB7‐Th as donor still has a little higher *V*_OC_ than that of devices based on COi6‐2Cl‐*δ*, and the device based‐COi6‐2Cl‐m received an intermediate *V*_OC_. The difference is that COi6‐2Cl‐m‐based devices achieved higher PCE than both the device‐based‐COi6‐2Cl‐*γ* and ‐COi6‐2Cl‐*δ*. To research the influence of mixing ratio on device performance, we carefully adjusted the ratios of COi6‐2Cl‐*γ* and COi6‐2Cl‐*δ*. The highest PCE of 9.30% was achieved when the ratio of COi6‐2Cl‐*γ* and COi6‐2Cl‐*δ* was 1:1.

##### Dichlorination

From the discussion of monochlorination, it can be seen that the introduction of Cl has a major influence on the energy levels, light absorption, crystallinity, and morphology of the compounds, mainly due to the strong electronegativity and empty 3d orbits of Cl which can accommodate *π*‐electrons. If the number of introduced chlorine atoms is increased, what will be the change in the effect of chlorination?

Hou et al. synthesized IT‐4Cl and made a comparison with IT‐2Cl (also known as Cl‐ITIC).^[^ ^19^ ^]^ Compared to ITIC, the introduction of Cl in both IT‐2Cl and IT‐4Cl has almost no effect on the optimal molecular conformations and the electron density distributions but brings obvious change to the HOMO and LUMO levels, which are ‐5.63 and ‐3.53 eV for IT‐2Cl, and ‐5.71 and ‐3.62 eV for IT‐4Cl, illustrating that with the increase of the number of Cl atoms from one to two in each of the end groups, both the HOMO and LUMO downshift further and the bandgap between HOMO and LUMO decreases, which explains the further redshift of the absorption spectrum of IT‐4Cl compared to IT‐2Cl. The addition of Cl does not bring conspicuous change to the optimal conformation, indicating that the addition of Cl to the end groups does not produce a significant additional steric hindrance effect, although Cl is much bigger than F. Both the absorption spectra of IT‐2Cl and IT‐4Cl are redshifted from solution to film, especially that of IT‐4Cl which is about 40 nm higher than that of IT‐4F, which was thought to be ascribable to the larger dipole moments of chlorinated molecules and also should be connected to their crystallinity in aggregation states. Both devices based on the two small molecules with PBDB‐T‐2F as donor obtained a PCE over 13%, respectively. IT‐4Cl‐based devices have an 80% EQE from 550 to 780 nm, contributing to the high *J*_SC_. But its *V*_OC_ is relatively low due to the low LUMO level. To increase the voltage, IT‐2Cl with a higher LUMO level was used together with IT‐4Cl to develop ternary solar cells. When the ratio of IT‐2Cl to IT‐4Cl is 2:8, a PCE of 14.18% is observed. Yan et al. compared the monochlorination and dichlorination of IXIC.^[^ ^62^ ^]^ The regularities of energy levels, light absorption and crystallinity are similar to those of the chlorinated derivatives of ITIC. However, there are some subtle differences. For example, IXIC‐2Cl and IXIC‐4Cl have the same HOMO energy level.

Introducing the dichlorinated end group to other acceptor cores also leads to excellent photovoltaic performances.^[^ ^62^, ^63^, ^64^, ^65^, ^66^, ^67^, ^68^ ^]^ Chen et al. designed a new small molecule acceptor, NCBDT‐4Cl, which blends with PBB‐T‐SF as the donor to give a high‐performance device with an extremely low *E*_loss_ of 0.55 eV.^[^ ^66^ ^]^ This as‐cast device could achieve a high PCE of 13.1% and 14.1% after optimization of solvent additive and thermal annealing. Another dichlorinated small molecule acceptor, IPIC‐4Cl, was also shown to enhance photovoltaic performances.^[^ ^65^ ^]^ By comparing with the nonhalogenated and fluorinated compounds, IPIC‐4Cl blending with PBDB‐T had higher crystallinity and better phase separation, ensuring efficient exciton dissociation and charge collection and achieving an outstanding PCE of 13.4% with an extremely low *E*_loss_ of 0.51 eV. Since PC_71_BM has been reported to function as the electron transport channel and solid processing‐aid, IPIC‐4Cl:PBDB‐T was applied to ternary solar cells using PC_71_BM as the third component, resulting in an excellent PCE of 14.3% and a high *J*_SC_ of 23.3 mA cm^−2^.

Y6, synthesized by Zou et al., has been a recently popular acceptor material.^[^ ^69^ ^]^ Yao et al. replaced the F atoms of Y6 and synthesized a chlorinated molecule, BTP‐4Cl, which showed a redshift of light absorption and reduced LUMO level.^[^ ^70^ ^]^ However, the device based BTP‐4Cl showed a higher *V*_oc_ compared to the device based Y6, due to the reduced nonradiative energy loss. Consequently, a high PCE of 16.5% was obtained with PBDB‐TF as donor. Then, the alkyl chain was tuned to further optimize the molecule.^[^ ^24^, ^71^ ^]^ First, they extended the alkyl chain on the pyrrole rings to improve the processability of BTP‐4Cl and found that the dodecyl was best for PCE as the alkyl chain on pyrrole rings (named BTP‐4Cl‐12).^[^ ^71^ ^]^ Our group also prepared the single crystal of the acceptor called BTIC‐BO‐4Cl, which showed short *π*–*π* distances (3.29–3.53 Å) between adjacent molecules because of multiple interlocked Cl^…^S and Cl^…^ *π* interactions. It formed a 3D network in its single crystal structure, leading to high electron mobility.^[^ ^72^ ^]^ Second, they shortened the alkyl chain to balance the relationship of between processability and device efficiency.^[^ ^24^ ^]^ As a result, they achieved an outstanding PCE of 17.8% by fabricating the device based the optimized molecule, BTP‐eC9, with PBDB‐TF as donor. BTP‐eC9:PBDB‐TF could also be processed by blade‐coating technology and the corresponding device with an area of 1 cm^2^ showed a high PCE of 16.2%.

**Figure 5:**
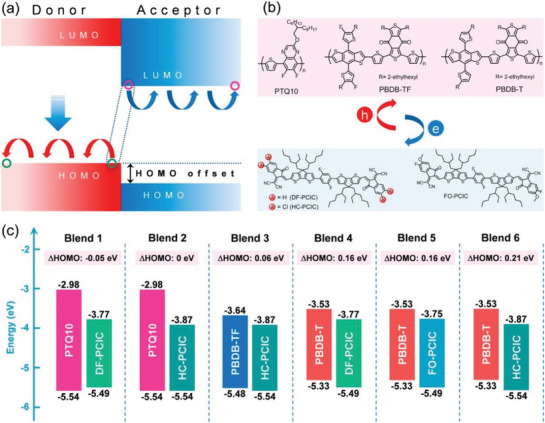
a) The working mechanism scheme of charge separation, b) molecular structures, c) energy levels of six different D:A blends with various HOMO offsets. Reproduced with permission.^[^ ^73^ ^]^ Copyright 2019, American Chemistry Society.

Chlorination also can help OSCs with a small driving force to work efficiently. Chen et al. designed a small molecule, HC‐PCIC, with dichlorinated IC end groups^[^ ^73^ ^]^ and compared it with the corresponding molecule without substituents and a structure with fluorine and methoxyl in each IC end. They observed that the chlorinated small molecule had more bathochromic‐shift and higher 0–0 peak, which implied the chlorinated compound has a stronger end‐to‐end arrangement because Cl has a larger size and dipole moment than H and F and improves the intermolecular interaction between the end groups. By matching the three small molecule acceptors and three polymer donors, a series of HOMO offsets were formed, as seen in **Figure** **5**. They found that the introduction of Cl helped the devices with small driving forces to perform efficiently, due to Cl strengthening the electron‐withdrawing parts. As a result, the HC‐PCIC‐based devices achieved two outstanding PCE of 10.42% and 11.75% with small HOMO offsets of 0 and 0.06 eV, respectively.

#### 3.2.2 Other Chlorinated End Groups

**Scheme 6:**
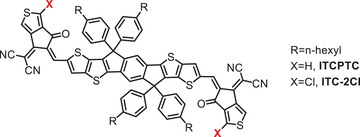
Small molecule acceptors based thiophene‐fused end groups.

Recently, IC end groups have been used in most A–D–A‐type small molecule acceptors. That few other effective end groups were reported may be because of the shortage of appropriate electron‐deficient active methylene precursors and synthetic difficulties, making it a difficult study.^[^ ^74^ ^]^ In 2017, Yang et al. fabricated a new active methylene compound, CPTCN (2‐(6‐oxo‐5,6‐dihydro‐4H‐ cyclopenta[c]thiophen‐4‐ylidene)malononitrile), using thiophene in place of the phenyl ring of INCN. With CPTCN as the precursor, they prepared a new ITIC analog, ITCPTC (see **Scheme** **6**), which displayed outstanding photovoltaic property owing to the suitable crystallinity and better film morphology.^[^ ^74^ ^]^ Later, they introduced a chlorine atom at the CPTCN end group and prepared a new chlorinated non‐fullerene acceptor named ITC‐2Cl.^[^ ^21^ ^]^ They found that the addition of the chlorine atom improved not only the electronic properties by bathochromic‐shifting the absorption spectrum and decreasing the LUMO, but also the molecular packing and thus the film morphology, which is similar to the regularity provided by a chlorinated IC end group. When the ITC‐2Cl was blended with PM6, the device obtained an impressive PCE of 13.6% (see **Table** **6**), with a lower *E*_loss_ of 0.67 V compared to the ITCPTC‐based device (PCE of 12.3% with *E*_loss_ of 0.70 eV) and even compared to the archetypal nonfullerene acceptors.

**Table 6:** Electronic and photovoltaic performance of small molecule acceptor based thiophene‐fused end groups

Acceptor	HOMO [eV]	LUMO [eV]	Donor	*V*_OC_ [V]	*J*_SC_ [mA cm^−2^]	FF [%]	PCE [%]
ITCPTC	−5.62	−3.96	PM6	0.95	17.6	73.4	12.3
ITC‐2Cl	−5.58	−4.01	PM6	0.91	20.1	74.1	13.6

#### 3.2.3 The Study of the Crystal Structure of Chlorinated NFAs

Besides the molecular structure, the crystal structure is also important in the photovoltaic performance. Chlorinated NFAs may have special crystal structures due to the unique characteristics of the chlorine atom with a large atom radius and the empty 3d orbits. Recently, in order to clarify the influence of chlorination on the stacking structures and further improve the photovoltaic performance, our group synthesized some chlorinated NFAs and cultivated their single crystals to gain a deep understanding of the relationship of structure and property.

In 2018, it was found that a chlorinated IC unit can effectively form interlocked molecular networks. Our group prepared IDIC‐4Cl, a small molecule acceptor, and found that it contained an intermolecular lock achieved by the Cl…S interactions and shown in the single crystal XRD analysis.^[^ ^67^ ^]^ The resulted networks not only led to a narrower and more ordered packing, but also gave rise to a better molecular coplanarity since the XRD showed that dihedral angles in the IDIC‐4Cl were very small and there was negligible torsion on the molecular backbone. The highly parallel IDIC‐4Cl molecular packing also led to a satisfactory J‐aggregation, which would be beneficial to charge transport to the electrodes. Hence unsurprisingly, devices based on IDIC‐4Cl and PBDB‐T2Cl obtained a high PCE of over 9.24%. Another case is R10‐4Cl, which has two different molecular orientations shown by the single‐crystal XRD analysis.^[^ ^75^ ^]^ One of the orientations is closely parallel packing with a *π*–*π* distance of 3.32 Å between the terminal groups of adjacent molecules. The other has the molecule obliquely inserted between those parallel molecules with their end groups interacting. An interpenetrated network was formed from these two orientations, benefiting the rapid charge carrier transfer both along the horizontal and the sloping direction. Hence, high charge carrier mobilities were achieved in the PBDB‐T:R10‐4Cl‐based devices, which had a PCE of 10.7%.

#### 3.2.4 The Application of Red‐Shift Absorption—Semitransparent OSCs

As shown above, chlorination of a small molecule acceptor always leads to red‐shift absorption, sometimes locating the main absorption in the near infrared (NIR) region. These NIR acceptors have great potential in the development of semitransparent OSCs.^[^ ^68^, ^76^, ^77^ ^]^

**Figure 6:**
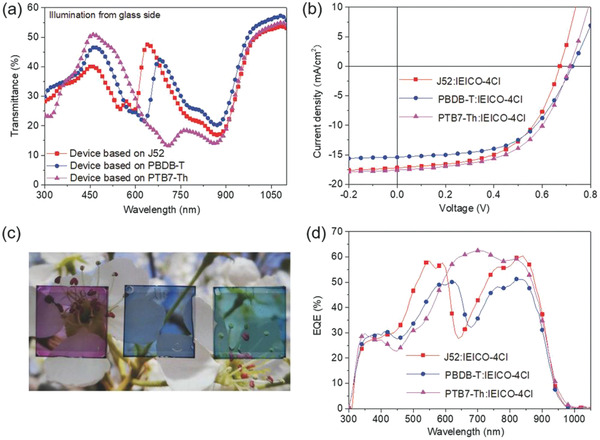
Transmission spectra a) and *J–V* curves b) of the semitransparent devices. c) Photograph of the three semitransparent devices, from left to right are J52: IEICO‐4Cl‐, PBDB‐T: IEICO‐4Cl‐, and PTB7‐Th: IEICO‐4Cl‐based devices. d) EQE curves of the semitransparent devices. Adapted with permission.^[^ ^63^ ^]^ Copyright 2017, Wiley.

In 2017, Hou et al. dichlorinated an IC unit to form IEICO‐4Cl, whose absorption spectrum is effectively redshifted by chlorination with the absorption onset shifting from 925 to about 1010 nm when compared to IEICO itself.^[^ ^78^ ^]^ It was noted that IEICO‐4Cl mainly absorbs light at a wavelength in the range of 745–945 nm, which is beyond the visible region and thus makes the molecule an excellent candidate to prepare semitransparent photovoltaic devices.^[^ ^63^ ^]^ Using Au as an electrode with which to study semitransparent OSCs, it was found by exploring the relationship between the thickness of the Au electrode and the AVT, that devices based on IEICO‐4Cl and PTB7‐Th with 30 nm Au could achieve a PCE of 8.28% and an average visible transmittance (AVT) of 25.7%. By cooperating IEICO‐4Cl with J52, PBDBT and PTB7‐Th as the donor respectively, blend films show a color from purple to blue to cyan (see **Figure** **6**), giving IEICO‐4Cl more potential to be applied for special use. This study demonstrates that chlorination is a useful tool to fine‐tune the materials’ energy levels and develop devices with different properties.

Subsequently, Li et al. synthesized a NIR‐absorbing nonfullerene acceptor, BT‐ClC, with four Cl at specific molecular sites.^[^ ^77^ ^]^ Compared to BT‐C, BT‐ClC shows a redshift of about 60 nm, which suggests increased IECT. In films, the absorption of BT‐CIC is mainly between *λ* = 650 and 1000 nm, indicating that it is transparent in the visible region and is very suited to the preparation of semitransparent OSCs. Devices in which BT‐ClC cooperates with PCE‐10 show a PCE of 11.2 ± 0.4%. The BT‐CIC‐based devices obtained a 75% EQE between 650 and 850 nm while having a transparency region between 400 and 650 nm. Using 10 nm Ag as a cathode, the semitransparent solar cells exhibited a PCE of 7.1% with an AVT around 43%. BT‐ClC implies that chlorination would be an excellent strategy with which to develop near‐infrared solar cells and high performance semitransparent solar cells.

Besides IEICO‐4Cl and BT‐ClC, use of many other chlorinated molecules, such as ITIC‐4Cl,^[^ ^68^ ^]^ ID‐4Cl,^[^ ^76^ ^]^ and ITC‐2Cl,^[^ ^21^ ^]^ has been reported. Such compounds can be used to prepare semitransparent solar cells and they achieve both high efficiency and transparency. Chlorination is therefore an effective strategy with which to prepare NIR materials. Compared to the corresponding unchlorinated molecules, the absorption spectra all redshift due to better IACT, and the absorption spectra redshift much more from solution to film due to stronger intermolecular interactions. Therefore, chlorination offers a new opportunity to obtained both high efficiency and transparency.

## 4 Main Characteristics of Chlorination: Comparison with Fluorination

From the introduction of the progress of chlorinated polymer donor and small molecule acceptors, it can be seen that the influence of chlorination is very complex. In this section, we try to extract the main characteristics of the chlorination and compare it to fluorination, seeking a deep understanding of both chlorination and fluorination.

### 4.1 The Yields and Cost of Chlorination and Fluorination

High yield and low cost are the main advantages of chlorination, which assigns chlorinated materials a high potential for commercial application. High yield and low cost mainly result from fewer synthetic steps, mild reaction conditions, and facile purification.^[^ ^20^, ^79^ ^]^ Incorporation of Cl‐containing organic semiconductors is much easier than with the F counterparts because the F‐containing molecules are usually prepared from other halogen‐containing precursors via exchange reactions,^[^ ^80^, ^81^, ^82^, ^83^ ^]^ which are generally operated under rigorous conditions and proceed with relatively low yields.

For example, the donor molecules PBDB‐T‐2Cl and PBDB‐T‐2F, which have very similar structures, except that PBDB‐T‐2F has fluorinated thiophene side chains, while the PBDB‐T‐2Cl side chains are chlorinated. Hou et al. carefully compared the synthetic routes of these two molecules.^[^ ^20^ ^]^ Both polymers were synthesized by the same polymerization method, but the two key precursors (compound 5, the precursor of PBDB‐T‐2F and compound 1, the precursor of PBDB‐T‐2Cl) of the two BDT‐T‐based monomers, were fabricated by different methods, which delivered different yields of the two polymers. Compound 1 could be easily prepared from 3‐chlorothiophene, an inexpensive chemical with yields between 75% and 85%. In contrast, the preparation of compound 5 uses four steps and is more complicated. These many steps led to a much lower overall yield of compound 5 of less than 15%. Moreover, compound 1 could be easily purified on a large scale by reduced pressure distillation, while compounds 3, 4, and 5 must be purified by column chromatography. Such purification must be accomplished on a small scale because the products’ Rf values in column chromatography are very close to those of both the starting molecules and the by‐products of each synthetic step. Thus, taken together, the lower costs of raw materials, the fewer synthetic steps and the easier purification of intermediates make the cost of synthesis of BDB‐T‐2Cl much lower than that of BDT‐T‐2F.

### 4.2 The Energy Level and Absorption

As strong electronegative elements, chlorine and fluorine have similar influence on energy levels, which is usually to decrease both the HOMO and LUMO, whether or not the chlorination and fluorination happened on donor materials such as donor units^[^ ^13^, ^33^ ^]^ or acceptor units^[^ ^84^ ^]^ or even *π*‐bridges,^[^ ^56^ ^]^ or acceptor materials.^[^ ^13^ ^]^ However, there are always exceptions.^[^ ^85^ ^]^ Because *V*_OC_ is decided by the gap between the HOMO of the donor and the LUMO of the acceptor, the deeper HOMO of donor materials will improve the *V*_OC_,^[^ ^47^ ^]^ while the deeper LUMO levels of acceptor materials will reduce it.^[^ ^65^ ^]^ The difference in the influence of the chlorination and fluorination on energy levels is termed the influence degree. Usually, the chlorination can deepen energy levels more efficiently, although the fluorine atom has the stronger electronegativity.^[^ ^67^ ^]^ The reason may be that the chlorine atom can accommodate *π*‐electrons more effectively. The empty 3d orbitals of Cl can accept any delocalization of *π*‐electrons, while F does not have energetically accessible empty orbitals for such delocalization.^[^ ^18^ ^]^ In addition, the small size of the fluorine atom can lead to severe electrostatic repulsion when *π*‐electrons are delocalized in its vicinity.^[^ ^18^ ^]^ Bao et al. used benzo[b]thiophene as a test system and his reported MO diagrams confirm that for both the HOMO and LUMO energy levels, the electron density of the F compound was enhanced over that of the Cl compound.^[^ ^18^ ^]^

Compared to the effect on energy levels, the influence of halogenation on light absorption is much more complex. Even though halogenation is in the same position of different molecules, such as the same difluorothiophene in PBDT[2F]T^[^ ^86^ ^]^ and PGeTFDTBT,^[^ ^87^ ^]^ the trends of the spectral shift are different. First, the situations of halogenation in copolymer acceptors will be discussed. Usually, the inductive electron‐withdrawing property of the F substitution leads to decreased electron density in the conjugated molecules and an increase in the HOMO–LUMO bandgap, consequently leading to the hypochromatic shift of the absorption spectra.^[^ ^88^, ^89^, ^90^ ^]^ In some cases, fluorination increases effective conjugation lengths induced by intramolecular F—H, F—F and/or F⋯S interactions, leading to a bathochromic shift.^[^ ^23^, ^91^, ^92^, ^93^, ^94^, ^95^, ^96^ ^]^ If both of these interactions exist in one molecule, the spectral shift may be the result of competition between them. Considering the molecular aggregation, fluorination may increase the intermolecular interaction leading to a bathochromic shift in solution,^[^ ^97^ ^]^ which can be confirmed by measuring the absorption spectra during a cooling process.^[^ ^98^ ^]^ According to the incomplete statistical results of reported works about fluorinated copolymer donors to date, majority fluorination leads to a blue‐shift^[^ ^12^, ^13^, ^84^, ^85^, ^86^, ^89^, ^90^, ^99^, ^100^, ^101^, ^102^, ^103^, ^104^, ^105^, ^106^, ^107^, ^108^, ^109^, ^110^, ^111^, ^112^, ^113^, ^114^, ^115^, ^116^, ^117^, ^118^ ^]^ and minority fluorination leads to a red‐shift.^[^ ^23^, ^87^, ^91^, ^94^, ^95^, ^96^, ^97^, ^98^, ^119^, ^120^ ^]^ As for the chlorination, according to the much fewer reported works, the trends are similar to those with fluorination. Majority chlorination leads to a blue‐shift^[^ ^32^, ^33^, ^48^ ^]^ and minority chlorination leads to a red‐shift.^[^ ^23^, ^56^ ^]^ However, the reasons are different. The increased steric hindrance is one of the reasons for the blue‐shift after chlorination.^[^ ^46^, ^48^ ^]^ The situation in small molecule acceptors with fluorinated or chlorinated end groups is much easier. Usually, the spectra of fluorinated or chlorinated molecules are redshifted compared to unhalogenated molecules according to the reported works.^[^ ^19^, ^21^, ^44^, ^59^, ^62^, ^65^, ^67^, ^77^, ^121^, ^122^, ^123^ ^]^ However, opinions about the reason for redshift diverge, and mainly include the enhanced IACT effect,^[^ ^67^ ^]^ the inductive effect and *π*‐electron delocalizing into empty d‐orbitals of the halogens,^[^ ^58^, ^124^ ^]^ and the larger dipole moments of Cl‐substitutions.^[^ ^19^ ^]^ Both chlorination and fluorination can enhance the extinction coefficients of both polymer donors^[^ ^13^, ^32^, ^34^, ^37^, ^106^ ^]^ and small molecule acceptors,^[^ ^21^, ^44^, ^59^, ^65^ ^]^ perhaps due to the hyperchromic effect of F and better packing.^[^ ^59^ ^]^

### 4.3 The Steric Hindrance and Morphology

Fluorine has a small van der Waals radius (1.47 Å), which leads to negligible steric effects, while the van der Waals radius of chlorine (1.76 Å) is much larger. Usually, fluorination can enhance the crystallinity due to the strong electronegativity supporting noncovalent intermolecular interactions, such as F···H and F···S. Combined with the small atom radius such interactions are subject to negligible steric hindrance. The situation with chlorination is more complex and varies with the position of chlorination, which decides the steric hindrance resulting in such a modification. If steric hindrance is significant, chlorination will distort the molecules and decrease the crystallinity. An example of this is Cl‐IIDT.^[^ ^23^ ^]^ If steric hindrance is avoided somehow, for example by chlorinating the end groups ^[^ ^62^, ^63^, ^64^, ^65^, ^66^, ^67^, ^68^ ^]^ and side chains,^[^ ^20^ ^]^ chlorination can increase the crystallinity due to strong noncovalent interactions, such as Cl···S and Cl···*π*. For conjugated materials, the dihedral angle between two adjacent conjugated units is one of the key factors influencing aggregation and morphology.^[^ ^23^, ^125^, ^126^ ^]^ Usually, if the chlorine is introduced directly onto the backbone of copolymers, the dihedral angle between two adjacent conjugated units will be increased.^[^ ^23^, ^49^ ^]^ For example, we found that in the copolymer of PBDTClBT, the dihedral angle between the monochlorinated BT and the Th (close to the chlorine) was 53.13°, while the opposite dihedral angle between monochlorinated BT and the Th (away from chlorine) was only 18.31°. The other dihedral angle between the Th (near the chlorine) and adjacent BDT unit was also only 20.05°.^[^ ^49^ ^]^ in another case, Pei et al. observed the strong preaggregation of F‐IIDT in solution owing to enhanced noncovalent interactions between the polymer backbones, leading to large crystal domains with no preferred crystallographic orientation, while the addition of a Cl atom enhanced the torsional angle of the molecular skeleton, leading to a decreased crystallization tendency and films with less crystallinity and face‐on molecular orientation, and consequently higher PCE of the Cl‐IIDT‐based device.^[^ ^23^ ^]^

**Figure 7:**
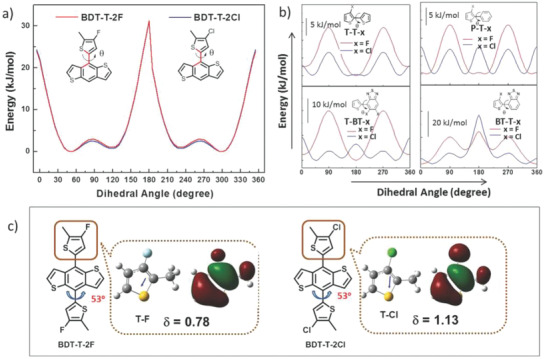
Theoretical calculation data. a) The twisting barriers of BDT‐T‐2F and BDT‐T‐2Cl; b) the twisting barriers of the four pairs of conjugated building blocks with F or Cl; c) the twisting effects on the two BDT units, the dipole moments and HOMO surfaces of thiophene side groups. Reproduced with permission.^[^ ^20^ ^]^ Copyright 2018, Wiley.

**Figure 8:**
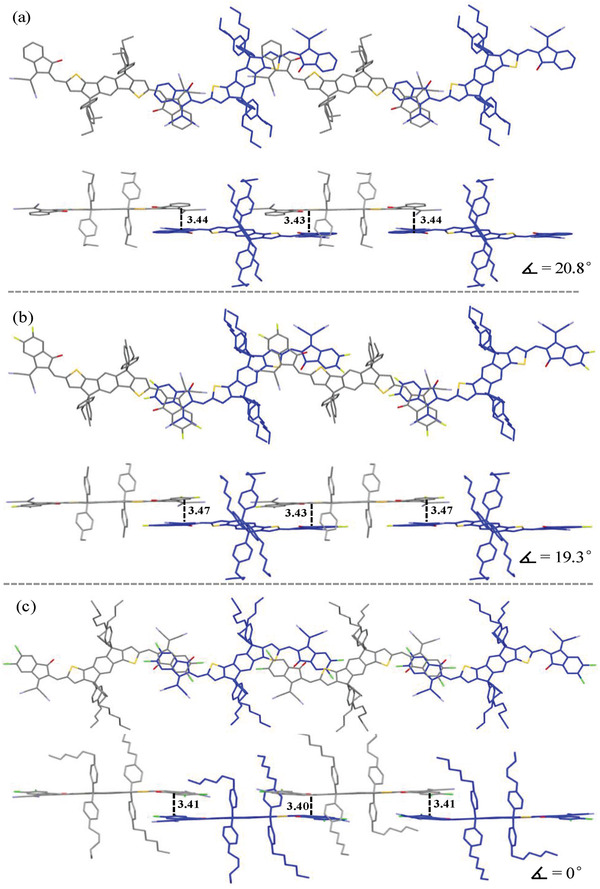
X‐ray crystallographic structures of a) IDIC‐4H, b) IDIC‐4F, and c) IDIC‐4Cl. (∡ represents the dihedral angles between IDT cores of adjacent molecules). Reproduced with permission.^[^ ^67^ ^]^ Copyright 2018, American Chemistry Society.

If other factors are not considered, the increase in the dihedral angle is adverse for photovoltaic performance, because it will break the coplanarity of the molecular backbone and damage the IACT, which is very important for D–A‐type organic semiconductors. Usually, there are two methods to avoid or reduce the influence of steric hindrance due to the introduction of chlorination. One is introduction of the chlorine atom onto the conjugated side chain. Hou et al. theoretically calculated the dihedral angle between fluorinated‐ or chlorinated‐Th side group and BDT unit and found the two dihedral angles are the same, 53° (see **Figure** **7**).^[^ ^20^ ^]^ Chen et al. found that when chlorinated, the Th side chain of the BDT unit in PBDT‐Cl‐3T2C, the dihedral angle between the conjugated side chain and backbone increased only a little, from 56.92° to 57.38°.^[^ ^35^ ^]^ The influence on the dihedral angle between adjacent units is therefore smaller. The other method is chlorinating the end group, which is usually done on small molecule acceptors.^[^ ^61^, ^67^ ^]^ Prepared by this method, chlorinated materials can have comparative or even more coplanar structures compared to the corresponding unchlorinated or fluorinated materials.^[^ ^60^, ^67^, ^127^, ^128^ ^]^ For example, Wei et al. speculated that the Cl‐substituted end group possesses a nearly coplanar geometry according to the single crystal of m‐ITIC‐OR‐4Cl, obtained by slow solvent vapor diffusion and analyzed by X‐ray crystallography.^[^ ^127^ ^]^ Hou et al. found the addition of Cl onto the end group did not bring distinct changes to the optimal conformation, implying that the addition of Cl onto the end groups did not produce additional steric hindrance.^[^ ^19^ ^]^ Our group has fabricated two new molecules, IDIC‐4Cl and IDIC‐4F, with Cl‐ and F‐substituted IC as terminal groups, respectively.^[^ ^67^ ^]^ To understand the influence of the addition of F and Cl on molecular configuration and packing, single crystals were cultivated and their X‐ray diffraction analysis was carried out. According to the data from single crystals, the dihedral angles between IDT cores and corresponding IC end groups of IDIC‐4H, IDIC‐4F, and IDIC‐4Cl were 14.9°, 16.3°, and 9.0°, respectively. Interestingly, the addition of F resulted in a small increase in the torsion, while the Cl‐substituted molecule showed a reduced dihedral angle, illustrating that the addition of Cl at the end groups will not induce a contorted *π*‐stacking molecular arrangement, in spite of the larger radius of Cl than that of F. Due to the strong Cl···S interactions between adjacent molecules, IDIC‐4Cl with an interlocked network configuration showed the shortest *π*–*π* distance of 3.41 Å, compared to the other two molecules (see **Figure** **8**). In addition, adjacent IDIC‐4Cl molecules are completely parallel, while the IDT cores of adjacent IDIC‐4H and IDIC‐4F have dihedral angles of 20.8° and 19.3°, respectively. The better coplanarity of IDIC‐4Cl molecules in one direction should be favorable for charge carrier transport between different connected molecules. The very small dihedral angles between adjacent chlorinated molecules were also observed by others.^[^ ^60^, ^128^ ^]^

## 5 The Synergistic Effect of Chlorination and Fluorination

Chlorination and fluorination are both efficient strategies to improve the PCE of OSCs and have different characteristics from one another. Therefore, introduction of a chlorine or fluorine atom in one molecule may combine the advantages of each atom. Some reported works have studied the synergistic effect of chlorination and fluorination.

The PTB family polymer with BDT and TT units is one of the most efficient classes of D–A polymers. Among the PTB family,^[^ ^26^, ^27^, ^28^, ^29^, ^30^, ^31^, ^32^, ^33^, ^34^, ^35^, ^36^, ^37^, ^38^, ^39^, ^40^, ^41^, ^42^, ^43^, ^44^, ^45^, ^46^, ^47^, ^48^, ^49^, ^50^, ^51^, ^52^, ^53^, ^54^, ^55^, ^56^, ^57^, ^58^, ^59^, ^60^, ^61^, ^62^, ^63^, ^64^, ^65^, ^66^, ^67^, ^68^, ^69^, ^70^, ^71^, ^72^, ^73^, ^74^, ^75^, ^76^, ^77^, ^78^, ^79^, ^80^, ^81^, ^82^, ^83^, ^84^, ^85^, ^86^, ^87^, ^88^, ^89^, ^90^, ^91^, ^92^, ^93^, ^94^, ^95^, ^96^, ^97^, ^98^, ^99^, ^100^, ^101^, ^102^, ^103^, ^104^, ^105^, ^106^, ^107^, ^108^, ^109^, ^110^, ^111^, ^112^, ^113^, ^114^, ^115^, ^116^, ^117^, ^118^, ^119^, ^120^, ^121^, ^122^, ^123^, ^124^, ^125^, ^126^, ^127^, ^128^, ^129^ ^]^ PTB7‐Th, made by adding the 2‐ethylhexyl‐thienyl group into the BDT to enhance the coplanarity of the main chain combined with keeping the F‐substituted TT with the 2‐ethylhexyl carboxylate group for lower HOMO,^[^ ^26^ ^]^ is one of the most efficient structures. To further decrease the HOMO level, we introduced a chlorine‐substituted TT (TT‐Cl) unit instead of fluorine‐substituted TT (TT‐F) obtaining PTBCl, and leading to an enhancement of *V*_OC_ from 0.80 to 0.91 V in the devices using ITIC as acceptor.^[^ ^47^ ^]^ However, when PBTCl is blended with the PC_71_BM as acceptor, the FF and *J*_SC_ are much lower than that of PTB7‐Th: PC_71_BM‐based device.^[^ ^46^ ^]^ This case shows the huge difference in the influence of chlorination and fluorination on the photovoltaic performance, although both chlorine and fluorine atoms have strong electronegativity. F, as a small atom with quite high electron affinity, can deepen the HOMO of molecules without deleterious steric effects. Unlike fluorine, chlorine atom can deepen the HOMO levels more efficiently. However, it may break the planarity of the conjugated polymer, which affects the morphology and photovoltaic performance, especially FF and *J*_SC_. In order to combine the advantages of chlorination and fluorination, our group synthesized a series of terpolymers, PBTCl*x*, by a triple‐component copolymerization method with a certain ratio of TT‐Cl and TT‐F units. By carefully controlling the number of TT‐Cl groups, we found that PBTCl25 with 25% TT‐Cl obtained the best PCE of 8.31%, which is attributed mainly to the enhanced *V*_OC_ from 0.79 to 0.82 V while retaining the optimized morphology and planarity of the conjugated molecule at the same time.

Our group also studied a different synergistic effect of chlorination and fluorination in another system. We synthesized PCBT4T‐2BO and PCBT4T‐2OD by introducing chlorine into the backbone at the position of the acceptor unit benthiadiazole and made PC_71_BM‐based bulk heterojunction PSCs of these polymers, which achieved enhanced *V*_OC_ and *J*_SC_. In this way, the PCE could be dramatically improved.^[^ ^52^ ^]^ Concerning tuning the energy levels, compared with fluorine atom, chlorine atom with a bigger atomic radius could reduce the energy levels more efficiently, thereby further improving the *V*_OC_ of the corresponding PSCs. We also synthesized monochloro and monofluoro‐substituted PCFBT4T‐2OD. The PSCs based on these constructs exhibited a further improved *J*_SC_ of 17.61 mA cm^−2^ and the highest PCE of 8.84%. The introduction of fluorine atoms into the polymer PCFBT4T‐2OD further enhanced the *π*–*π* stacking, compared with the one‐chlorine substituted PCBT4T‐2BO, which was helpful for the charge transport in the active layer and enhancement of the device performance in PSCs. These results implied that the synergistic effect of chlorination and fluorination may be an effective molecular design strategy for efficient PSCs.

## 6 Conclusion

In this review, we have introduced some of the intrinsic properties of the chlorine atom and compared them with that of fluorine atom, followed by the performance of chlorinated polymer donors and small molecule acceptors. A chlorine atom has strong Pauling electronegativity (slightly lower than that of the fluorine atom) and empty 3d orbitals, which can accept the electron pairs and *π* electrons and can help to delocalize electrons, due to which chlorination can efficiently downshift the energy levels, even more efficient than fluorination; a chlorine atom has a large van der Waals radius leading to larger steric hindrance, which can be avoided by thoughtful structural design; the C—Cl bond has a large dipole moment, which expands the absorption range, deepens the molecular energy levels and improves molecular stacking, which is also beneficial to expand the absorption. Chlorination has a deep influence on the photophysical and photovoltaic properties, and usually decreases both the HOMO and LUMO simultaneously in both donor and acceptor molecules. Compared to the effect on energy levels, the influence of chlorination on light absorption is much more complex. For polymer donors, the chlorination mainly led to a blue‐shift while very little chlorination led to a red‐shift. For the small molecule acceptor, the spectra of chlorinated molecules are usually redshifted. The results of comparing the final photovoltaic performance of fluorinated and chlorinated materials are different case by case. Therefore, we can choose a proper strategy according to different situations. However, considering the high yields and low cost, chlorination has very promising potential for commercial applications.

## Conflict of Interest

The authors declare no conflict of interest.
